# Neutropenic Mice Provide Insight into the Role of Skin-Infiltrating Neutrophils in the Host Protective Immunity against Filarial Infective Larvae

**DOI:** 10.1371/journal.pntd.0004605

**Published:** 2016-04-25

**Authors:** Nicolas Pionnier, Emilie Brotin, Gregory Karadjian, Patrice Hemon, Françoise Gaudin-Nomé, Nathaly Vallarino-Lhermitte, Adélaïde Nieguitsila, Frédéric Fercoq, Marie-Laure Aknin, Viviana Marin-Esteban, Sylvie Chollet-Martin, Géraldine Schlecht-Louf, Françoise Bachelerie, Coralie Martin

**Affiliations:** 1 Unité Molécules de Communication et Adaptation des Microorganismes (MCAM, UMR 7245), Sorbonne Universités, Muséum National d’Histoire Naturelle, CNRS; CP52, Paris, France; 2 UMR996—Inflammation, Chemokines and Immunopathology, Inserm, Univ Paris-Sud, Université Paris-Saclay, Clamart and Châtenay-Malabry, France; 3 US31-UMS3679 -Plateforme PLAIMMO, Institut Paris-Saclay d’Innovation Thérapeutique (IPSIT), Inserm, CNRS, Univ Paris-Sud, Université Paris-Saclay, Clamart, France; University of Manchester, UNITED KINGDOM

## Abstract

Our knowledge and control of the pathogenesis induced by the filariae remain limited due to experimental obstacles presented by parasitic nematode biology and the lack of selective prophylactic or curative drugs. Here we thought to investigate the role of neutrophils in the host innate immune response to the infection caused by the *Litomosoides sigmodontis* murine model of human filariasis using mice harboring a gain-of-function mutation of the chemokine receptor CXCR4 and characterized by a profound blood neutropenia (Cxcr4^+/1013^). We provided manifold evidence emphasizing the major role of neutrophils in the control of the early stages of infection occurring in the skin. Firstly, we uncovered that the filarial parasitic success was dramatically decreased in Cxcr4^+/1013^ mice upon subcutaneous delivery of the infective stages of filariae (infective larvae, L3). This protection was linked to a larger number of neutrophils constitutively present in the skin of the mutant mice herein characterized as compared to wild type (wt) mice. Indeed, the parasitic success in Cxcr4^+/1013^ mice was normalized either upon depleting neutrophils, including the pool in the skin, or bypassing the skin *via* the intravenous infection of L3. Second, extending these observations to wt mice we found that subcutaneous delivery of L3 elicited an increase of neutrophils in the skin. Finally, living L3 larvae were able to promote in both wt and mutant mice, an oxidative burst response and the release of neutrophil extracellular traps (NET). This response of neutrophils, which is adapted to the large size of the L3 infective stages, likely directly contributes to the anti-parasitic strategies implemented by the host. Collectively, our results are demonstrating the contribution of neutrophils in early anti-filarial host responses through their capacity to undertake different anti-filarial strategies such as oxidative burst, degranulation and NETosis.

## Author Summary

Filariases are chronic debilitating diseases caused by parasitic nematodes affecting more than 150 million people worldwide. None of the current drugs are selective, neither able to eliminate the parasites nor to prevent new infections once the drug pressure has waned. Therefore, blocking the entry and the migration of the infective larvae (L3) could be an efficient way to control the infection. In the present study we investigated the early interaction between the host and the *L*. *sigmodontis* murine filariasis with a focus on the neutrophils in the innate host responses. We uncovered a key role of neutrophils in the control of infection provided by the CXCR4-gain-of-function mice (Cxcr4^+/1013^) that display a blood neutropenia as well as an accumulation of skin-infiltrating neutrophils. Overall, we reveal that in the early phase of filariasis, i.e. after L3 are delivered into the skin and before they reach their site for reproduction, neutrophils are critical elements of the host innate protective response arsenal. A better understanding of their indirect and/or effector role(s) may provide mechanistic clues to host factors implicated in parasitic nematode entry and potentially lead to the identification of new drug targets.

## Introduction

Filarial nematodes constitute a large group of human pathogens (*i*.*e*. *Onchocerca volvulus*, *Brugia malayi*, *Brugia timori*, *Wuchereria bancrofti*, *Loa loa*, *Mansonella spp*.) infecting around 150 million people throughout the tropics with more than 1.5 billion at risk of infection. Filariases remain a health issue, as no selective and efficient treatments are able to prevent and eliminate filarial infections in the exposed and infected populations [1].

Early stages of infection are common to filarial nematodes with the infective stages of filariae (L3) being delivered in the skin of the host by blood feeding arthropods [2]. However later in their life cycle, depending on the filarial species, adult stages settle in their preferred tissues for maturation/reproduction/release of microfilariae, strongly suggesting different migration paths for the larvae. Consequently, adult filariae reside in connective tissues (*i*.*e*. dermis, subcutaneous tissue, aponeuroses, tendons), blood and lymphatic vessels, or coelomic cavities such as the pleural cavity in the case of the rodent filarial *L*. *sigmodontis* [3], which is the focus of our study.

*L*. *sigmodontis* is a widely used experimental model in which infective larvae migrate from the skin through the lymphatic system before ending up in the pleural cavity [4]. Almost 70–80% of the skin-inoculated L3 will not reach their maturation niches [4, 5], supportive of early defense mechanisms implemented in the skin by the mice. The innate immune response to nematodes can involve host cell populations such as eosinophils, neutrophils, mast cells and activated macrophages [6–9]. A clear consensus has emerged from the *L*. *sigmodontis* model regarding the role of eosinophils in the early protective immunity against L3, induced within two days in infected mice immunized with irradiated L3 [10–13]. Several lines of evidence from the various filarial models including *L*. *sigmodontis* also indicate that neutrophils might contribute to the host immunity, but at late stages of the infection notably through the control of the adult worms and blood-circulating microfilariae [14–16]. Although it has not been reported that an innate immune response dominated by neutrophils might kill incoming L3, neutrophils are recruited to sites of parasitic nematode entry in *Nippostrongylus brasiliensis*, *Heligmosomoides polygyrus*, *Brugia pahangi* [17–20], and *L*. *sigmodontis* infections [12]. Additionally, neutrophils were reported to contribute to macrophage-dependent resistance mechanisms against *Strongyloides stercoralis* and macrophages-dependent resolution of tissue damage induced by *Nippostrongylus brasiliensis* [21–23]. Moreover, along with their essential role in responses to microbial pathogens, neutrophil activation and recruitment could be attributed to the endobacteria *Wolbachia* harbored by some filarial parasites [24–26]. Interestingly, the resistance mechanisms of the host in early defense against L3 also target these symbionts [26, 27].

Neutrophil homeostasis, which is maintained by a balance between granulopoiesis in the bone marrow (BM) and migration between blood and tissues, is finely tuned by the chemokine system. The essential CXCL12/CXCR4 chemokine-chemokine receptor pair [28–30], which induces typical activation of the Gαi protein- and β-arrestin-dependent pathways [31–33] regulates with other chemokine receptors, hematopoiesis and the lymphoid and peripheral trafficking of neutrophil and lymphocyte subsets [33]. The CXCL12/CXCR4 axis is notably critical for the release of neutrophils from the BM [34] and lungs [35]. CXCR4 engagement also promotes the migration of neutrophils from inflamed skin into draining lymph nodes, a process thought to participate in the control of pathogens through initiation of immune responses or conversely in the spreading of infection [36–38].

We have previously shown that a CXCL12-dependent cell response is associated with the “resistant phenotype” of C57BL/6 mice to *L*. *sigmodontis* infection [39] raising the possibility that this chemokine participates in the host immune-mediated resistance mechanisms. Here we thought to examine this possibility in C57BL/6 mutant mice, which harbor an inherited heterozygous *Cxcr4* mutation engendering a gain-of-CXCR4 function [Cxcr4^+/1013^], an anomaly linked to the rare immunodeficiency WHIM disorder [40, 41]. As a consequence, [Cxcr4^+/1013^] mice exhibit a peripheral leukopenia affecting blood lymphocytes and neutrophils as do patients. This leukopenia is transiently reversed in mice [41] and patients [42] upon inhibition of CXCR4 with the selective antagonist AMD3100 thus demonstrating the causal role of the gain-of-CXCR4 function. Interestingly, neutropenia of the mutant Cxcr4^+/1013^ mice occurs in the context of normal maturation of the granulocyte lineage [41] in support of the fact that transient normalization of blood counts upon CXCR4 blockade or patient’s infections [43] likely arises from disturbed neutrophil trafficking rather than from a production defect [41].

Our findings reveal a dramatic blockade of *L*. *sigmodontis* infection in mutant mice, which manifests early in the skin of mice inoculated with infective L3 and is related to a higher number of neutrophils in the skin of mutant mice as compared to their wt counterpart. The identified host-resistance mechanisms such as the increase of neutrophils infiltrating the skin, the oxidative burst response, and the release of NET promoted by L3 were extended to wt mice demonstrating that neutrophils are important contributors of the early host resistance mechanisms against the nematode.

## Results

### Drastic reduction of the filarial load in Cxcr4^+/1013^ mice

**Fig 1:**
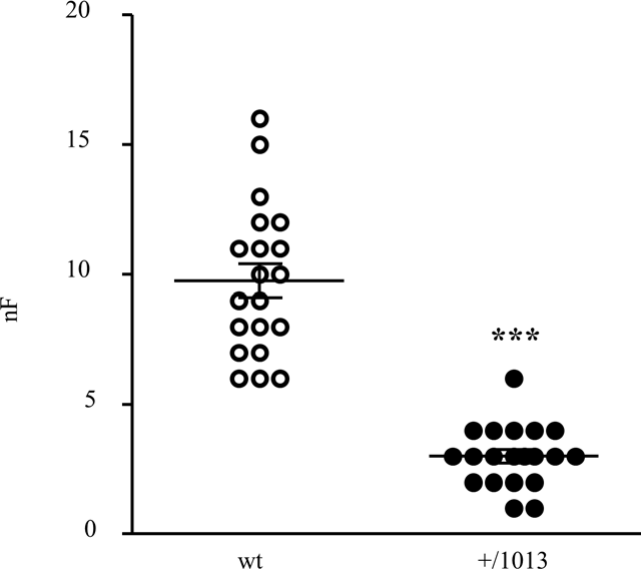
Parasitic success is dramatically reduced in Cxcr4^+/1013^ mutant mice. *L*. *sigmodontis* (40 larvae) were subcutaneously injected into wt and Cxcr4^+/1013^ C57BL/6 mice. Worms were harvested in the pleural cavity of the mice 20 days p.i. and counted (number of L3 filariae, nF). Results are expressed as mean +/- SEM (9.75 +/- 0.65 and 3 +/- 0.26 for wt and Cxcr4^+/1013^ C57BL/6 mice respectively), n = 20 and each mouse is represented by a dot (pool of 4 independent experiments with 5 mice per group), t-test ***: p < 0.001.

Infective larvae were subcutaneously injected into wild type (wt) and Cxcr4^+/1013^ C57BL/6 mice and were recovered in the pleural cavity 20 days post-inoculation (p.i.), when the filarial load is still high and before it decreases, as observed in the resistant background of the mice (S1 Fig). The mean number of worms recovered from the wt mice (9.72 ± 0.65 SEM, Fig 1) was similar to the one previously reported for resistant C57BL/6 mice at this stage of the infection [44–47]. As compared, the mean filarial load dramatically dropped in the mutant mice to minute levels (3 ± 0.26 SEM) corresponding to a 70% decrease in the filarial parasites (Fig 1). An enumeration performed earlier at day 8 p.i., after arrival of most of the L3 larvae in wt mice pleural cavity (11 ± 0.5 SEM), indicated a similarly small number of worms recovered from the mutant mice (4.4 ± 0.7 SEM) (S2 Fig). These results excluded the possibility that a killing of L3 larvae in the pleural cavity of mutant mice may account for the decreased filarial recovery in these mice.

### Filarial infection promoted reversion of neutropenia in Cxcr4^+/1013^ mice

**Fig 2:**
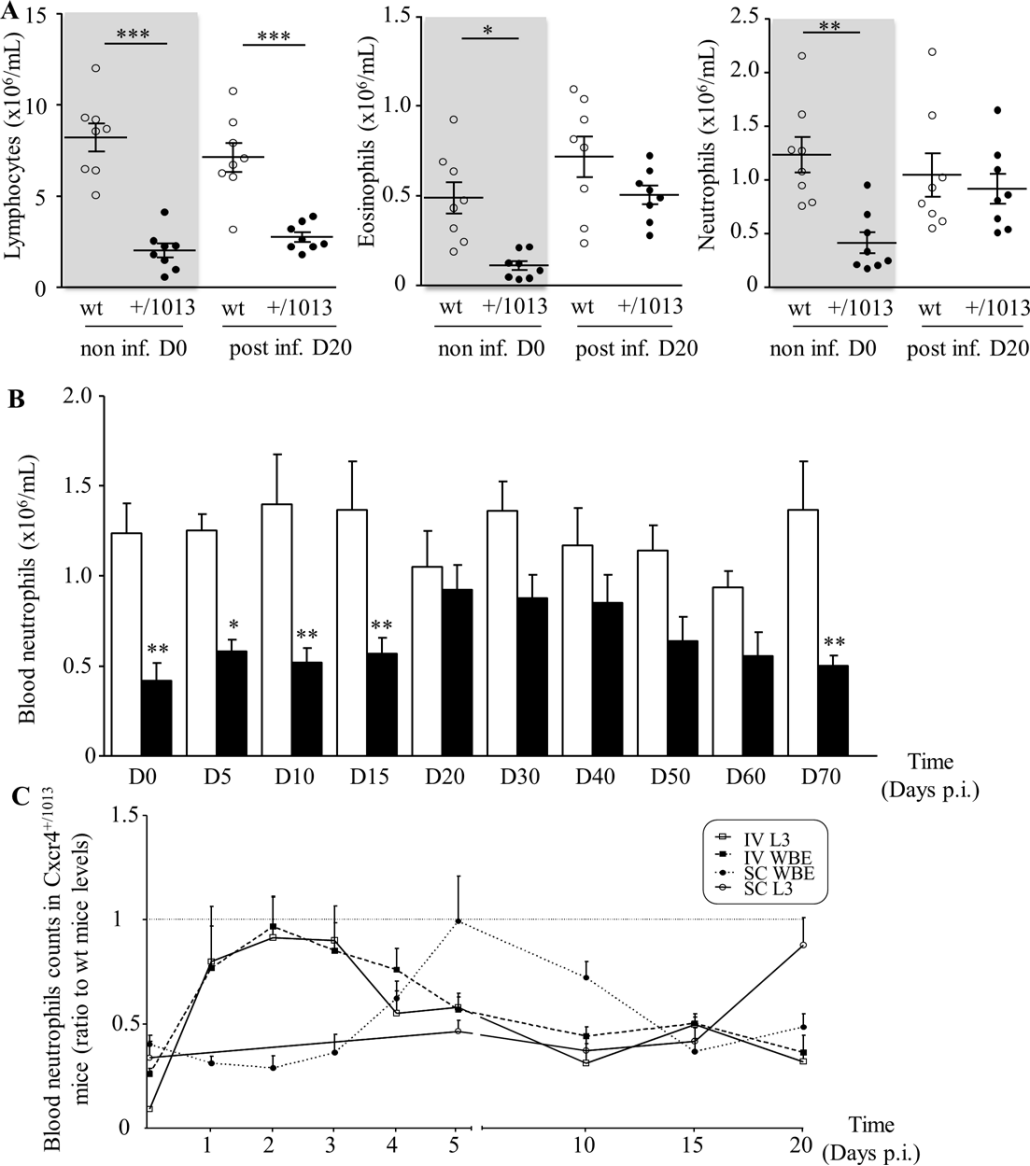
Transient reversion of neutropenia in infected Cxcr4^+/1013^ mice. A: Total numbers of lymphocytes, eosinophils and neutrophils were determined on blood smears from non-infected mice at day 0 (non inf. D0) and from subcutaneously infected mice 20 days p.i. (post inf. D20). Open dots and black dots are representing wt and Cxcr4^+/1013^ mice, respectively (n = 8, each mouse is represented by a dot). Means +/- SEM were at D0 for wt and Cxcr4^+/1013^, respectively: for lymphocytes 8.21 +/- 0.77 and 2.02 +/- 0.39; for eosinophils, 0.49 +/- 0.09 and 0.11 +/- 0.03 and for neutrophils 1.24 +/- 0.17 and 0.42 +/- 0.1. Differences between wt and Cxcr4^+/1013^ mice were analyzed by t-test. *: p < 0.05, **: p < 0.01, ***: p <0.001. B: Total numbers of blood neutrophils throughout the course of the filarial infection in infected wt and Cxcr4^+/1013^ mice (n = 8). Differences between wt and Cxcr4^+/1013^ mice were analyzed by one-way ANOVA with repeated measures then Bonferroni. *: p < 0.05, **: p < 0.01. C: Blood neutrophils counts in Cxcr4^+/1013^ mice at different time points upon SC inoculation with either 40 infective larvae (SC L3, ○, plain line) or 10μg L3-derived whole body crude extracts (SC WBE, ●, dotted line) or upon IV inoculation with 40 infective larvae (IVL3, Ƴ, plain line) or 10μg L3-derived whole body crude extracts (IVWBE, ■, dotted line). Neutrophil counts were done over a 20 days’ time period following inoculation and were expressed as ratio to wt mice levels. Ratios close to 1 depict a trend toward normalization of the blood neutrophils counts in the Cxcr4^+/1013^ mice. Raw data are presented in S2 Table. Results are expressed as mean + SEM, n = 4 to 5 and 8.

Upon arrival of the L3 in the pleural cavity, mice mount a cellular response directed against the worms, which reaches a peak 30 days p.i. when larvae are molting into young adults [39, 46, 48]. In this context, resistant C57BL/6 mice are characterized by having a higher increase of pleural exudate cells (PleCs) as compared to susceptible mice [39]. PleCs counts were increased upon infection in both type of mice but neither the levels nor the composition was significantly different between control wt and Cxcr4^+/1013^ mice (S3 Fig) at steady state and upon infection. These results are suggesting that the strongest resistance of mutant mice to filarial infection was not associated with any change in PleCs. We then investigated whether the leukocyte populations in the blood circulation were differentially affected in the mutant mice upon infection. Lymphocytes counts of wt and mutant animals were not significantly modified by 20 days post infection and the mutant mice remained lymphopenic (Fig 2A, left panel) throughout the infection up to 70 days p.i. In contrast, both eosinophil and neutrophils blood counts, also constitutively diminished in mutant mice, were almost normalized 20 days p.i. (Fig 2A, middle and right panels). Time course analysis of the circulating neutrophils counts in Cxcr4^+/1013^ mice throughout subcutaneous (SC) infection of L3 revealed an increase, which tended to normalize the circulating neutrophil counts from 20 days followed by a progressive decay back to initial levels at day 70 p.i. (Fig 2B). While a slight increase of blood neutrophil counts was observed in the first five days following injection (days 1–5), the levels remained significantly different between both wt and mutant mice (see S1 Table).

We further investigated further whether this late and sustained neutrophilia was related to infection as an indirect consequence of the SC delivery of L3 and/or directly *via* filarial immunomodulatory molecules present in whole body crude extracts (WBE). To do so we counted blood neutrophils in Cxcr4^+/1013^ mice throughout 20 days p.i. in four different experimental settings: inoculation of L3 (L3) versus L3 crude extracts (WBE) and SC inoculation compared to intravenous (IV) one. Neutrophils counts in Cxcr4^+/1013^ mice shown in Fig 2C were expressed as a ratio to wt mice counts, in order to illustrate the trend toward normalization (raw data are presented in S2 Table). Inoculation of L3 in Cxcr4^+/1013^ mice *via* the IV route (IV L3) was associated with an increase in circulating neutrophils, the levels of which tended toward a transient normalization between days 1 and 3 p.i. A similar response was observed upon IV inoculation of L3 crude extracts (IV WBE) indicating that this increase is independent upon the mobility of the larvae. As previously shown, SC injection with infective larvae (SC L3) promoted a long-lasting normalization of neutrophils counts at 20 days p.i., which persisted for at least 40 days (Fig 2B and 2C). SC inoculation of crude extracts (SC WBE) only promoted a transient normalization, which occurred much earlier at 5 days p.i. (Fig 2C). Altogether these results indicated that L3 either through immunomodulatory molecules (WBE) and/or their motility have, independently of the inoculation site, the potency to induce a transient neutrophilia in the mutant mice reaching neutrophils counts comparable to those in wt mice. However long-term normalization of blood neutrophil counts in the mutant mice over is strictly dependent upon the inoculation of live larvae into the skin.

### Bypassing the skin migratory phase strongly increased filarial success in both wt and Cxcr4^+/1013^ mice

**Fig 3:**
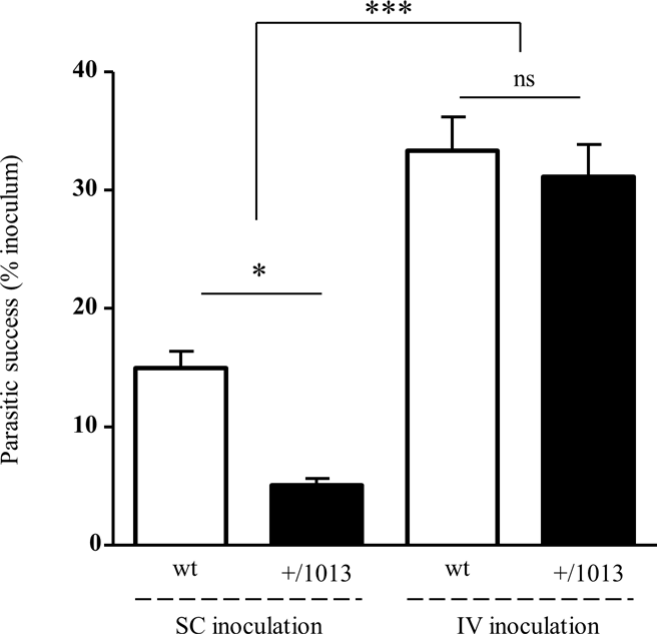
Reversion of the parasitic success in Cxcr4^+/1013^ mice upon intravenous inoculation. Both wt (white bars) and Cxcr4^+/1013^ (black bars) mice were injected with 40 infective larvae either subcutaneously (SC) or intravenously (IV) and worms were recovered in the pleural cavity at 20 days p.i. Filarial loads (means +/- SEM) were 5.89 +/- 0.53 and 2 +/- 0.21 for the wt and Cxcr4^+/1013^ SC-inoculated mice, respectively; and 13.1 +/- 1.1 and 12.25 +/- 1.1 for the wt and Cxcr4^+/1013^ IV-inoculated mice, respectively. The parasitic success represents for each mouse the larvae counts recovered in the pleural cavity expressed as percent of the inoculum. Results are expressed as mean +/- SEM, n = 7 to 12, One-way ANOVA then Bonferroni *: p < 0.05; ***: p < 0.001; ns = not significant.

We then compared the parasitic success upon IV and SC inoculation of Cxcr4^+/1013^ mice at 20 days p.i. Interestingly, we unraveled that upon IV inoculation filarial larvae also ended up in the pleural cavity. Strikingly, IV inoculation reversed the resistant phenotype of Cxcr4^+/1013^ mice observed upon SC inoculation (Fig 1) with a recovery of larvae in the pleural cavity reaching the levels obtained in the wt mice (Fig 3). Moreover this increase of the parasitic success was extended to wt mice, which displayed twice more filariae recovered in the pleural cavity upon IV inoculation as compared to SC inoculation (Fig 3). Collectively, these data support on one hand the concept that L3 migration from the skin to the pleural cavity would stand a blood stage. On the other hand, they strongly suggest that the mechanisms underlying the enhanced resistance of the Cxcr4^+/1013^ mice to infection are acting before larvae reach the bloodstream, and more generally bring evidence in support of the skin’s major role in the host defense against filariae.

### Neutrophils numbers are constitutively elevated in the skin of Cxcr4^+/1013^ mice

**Fig 4:**
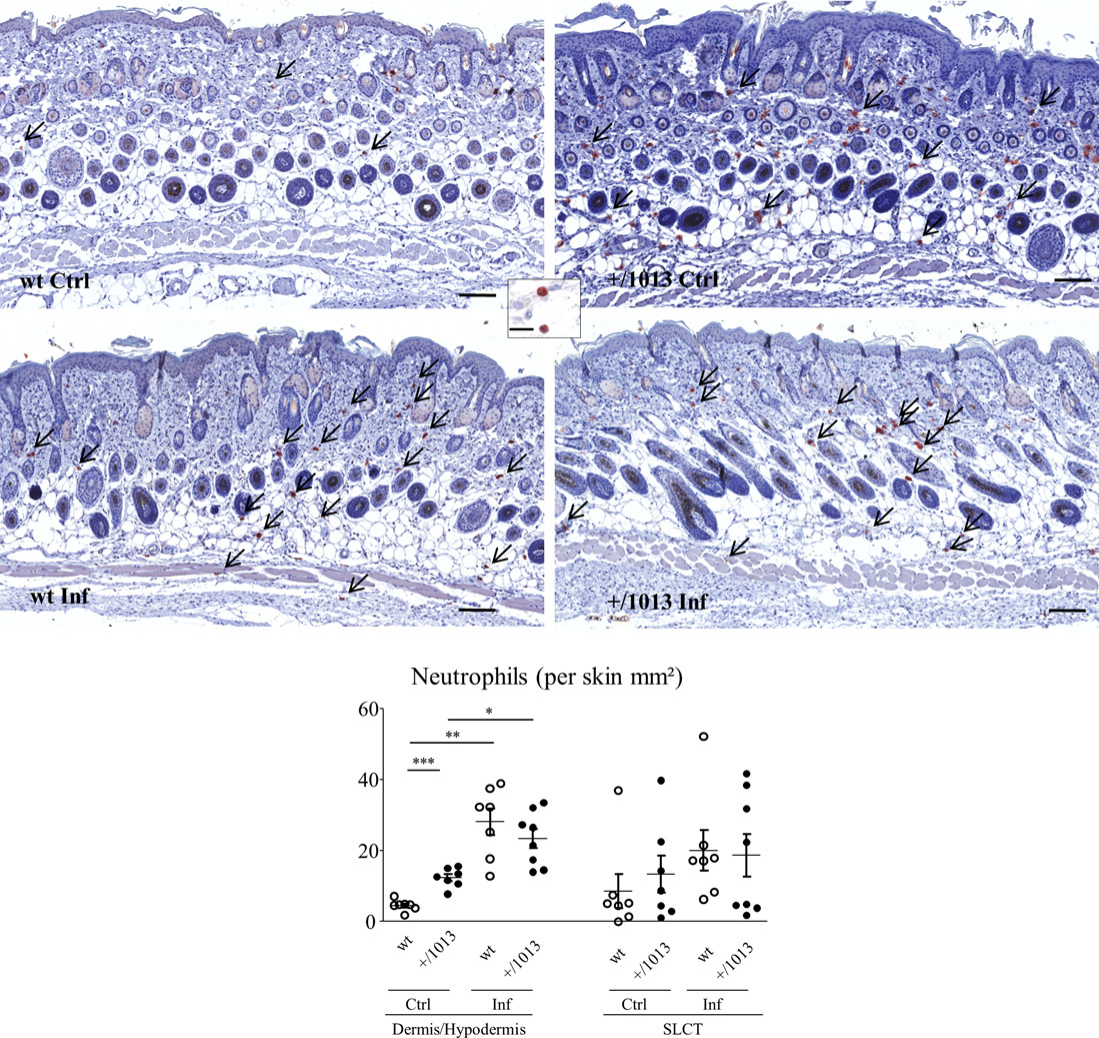
Steady state levels and recruitment of neutrophils in the skin upon filariae inoculation. Skin sections were collected before inoculation (control mice, Ctrl) or excised from the inoculation site 6 hours p.i. (Infected mice, Inf), embedded in paraffin and stained with anti-NIMP-R14 for the visualization of neutrophils. Representative H&E stained skin sections for each group (wt and Cxcr4^+/1013^ control (Ctrl) and infected mice (Inf)) are displayed and the total number of neutrophils per mm^2^ skin in the dermis-hypodermis and the SLCT layers are given in the graph below. Representative sections: scale bar = 100μm, magnification x40. Representative x100 magnifications: scale bar = 40μm. n = 7 to 8 per group. One-way ANOVA then Bonferroni *: p < 0.05; **: p < 0.01, ***: p < 0.001.

We thought to compare the steady state amounts of eosinophils, macrophages, mast cells and neutrophils in the skin of wt or mutant mice to those reached in both mice upon 6 hours p.i. considering the potential role of these cells in parasite clearance at early stages of the infection. The constitutive levels of eosinophils were similar in the skin of control wt and Cxcr4^+/1013^ mice whatever the layer either the dermis/hypodermis or the subcutaneous loose connective tissue (SLCT). Additionally, upon infection, eosinophil levels were similarly increased (5-fold) in both layers of the mutant and wt mice (S4A Fig). The numbers of macrophages and mast cells, degranulated or not, were also found to be in the same range in Cxcr4^+/1013^ and wt mice, but not significantly increased upon infection in any of the two models (S4B and S4C Fig). In contrast, the number of resident neutrophils constitutively present in the dermis-hypodermis of Cxcr4^+/1013^ mice was significantly higher than in wt mice (12.4 ± 1 SEM vs 4.79 ± 0.6 SEM neutrophils per mm^2^ of skin respectively, p < 0.001) (Fig 4). While an increasing trend was also observed in SLC layer, it was not significant. Filarial infection promoted a significant recruitment of neutrophils in the dermis/hypodermis of both mice, resulting in comparable levels of neutrophils at 6 hours p.i. in Cxcr4^+/1013^ and wt mice (28 ± 3.7 SEM and 23.4 ± 2.7 SEM neutrophils per mm^2^ of skin respectively). Cxcl12, which is expressed in the dermal stroma [49, 50], was found at higher levels in mutant mice dermis as compared to their wt counterparts (S5 Fig). Additionally, Cxcl12 levels were markedly increased in the dermis of both Cxcr4^+/1013^ and wt mice 6 hours p.i. (S5 Fig). Thus the steady state levels of the chemokine and their increase after infection are mirroring the variation in dermal neutrophil numbers herein reported in mice strengthening the possible interplay between both processes. Significantly, this increase in skin neutrophil recruitment observed in the 6 hours following filarial infection was concomitant with an increase of blood neutrophil numbers in both wt and Cxcr4^+/1013^ mice, of 2.9 and 2.7 fold respectively (S6 Fig). This phenomenon observed both in mutant and wt mice is highly suggestive of a neutrophil release in the bloodstream triggered upon immune sensing of the filariae by skin-resident cells.

### Neutrophils are key players in the protection against filariae in Cxcr4^+/1013^ mice

**Fig 5:**
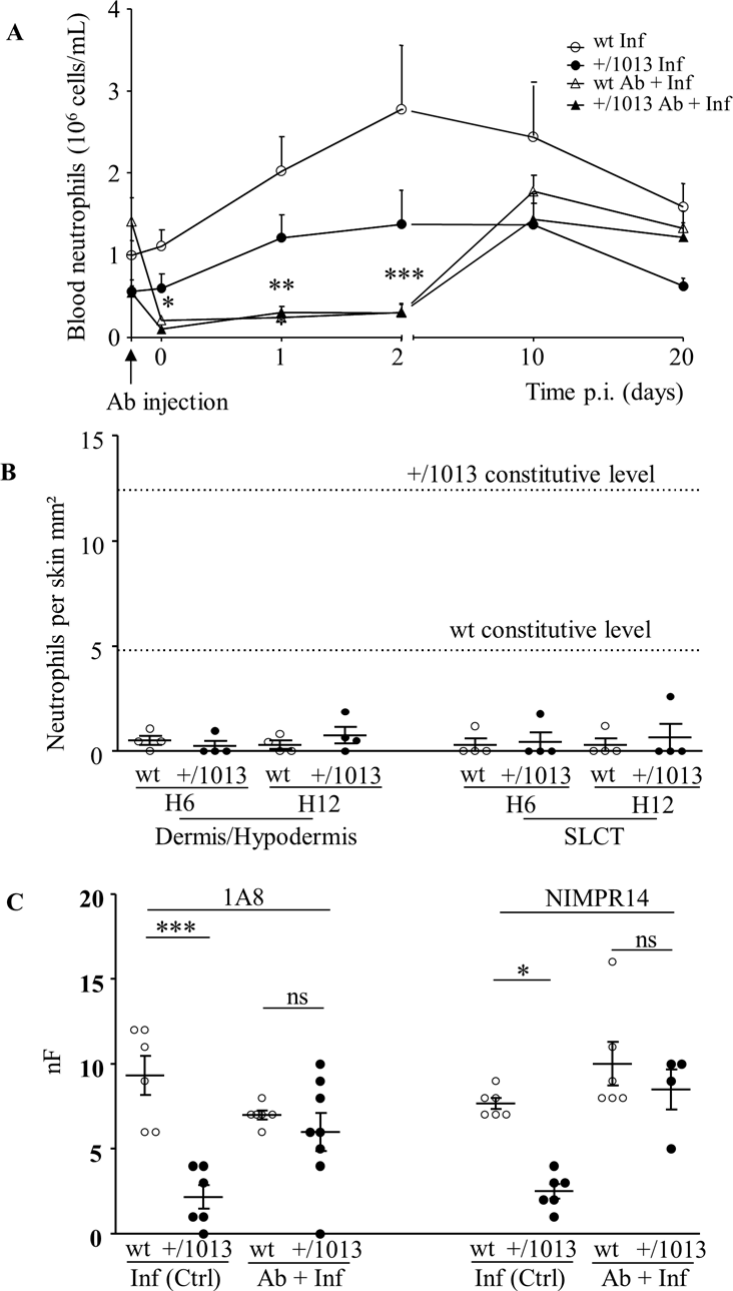
Effect of neutrophil depletion on parasitic burden. Neutrophil depletion was performed via intraperitoneal inoculation of anti-Ly6G antibody 6 hours (A, arrow) prior SC inoculation with filariae (Day (D) 0) and analyses were carried out at the indicated time throughout the course of the filarial infection up to 20 days. A and B: Effect of the neutrophil-depleting treatment on circulating neutrophil levels (A) and skin-resident neutrophils (B). A: Blood levels of neutrophils from the onset of the inoculation of the anti-Ly6G (clone 1A8) antibody 6 hours prior SC infection up to 20 days p.i. in wt (open triangles) and Cxcr4^+/1013^ (black triangles) mice. Control wt (open dots) and Cxcr4^+/1013^ mice (black dots) were injected with PBS. Results are expressed as mean +/- SEM, n = 6 to 8, One-way ANOVA p < 0.05, Bonferroni *: p < 0.05, **p < 0.01, ***: p < 0.001. B: Effect of the anti-Ly6G (clone 1A8) antibody on skin-infiltrating neutrophils 6 hours (H6) and 12 ous (H12) after antibody injection in non-infected mice. Skin sections were stained with anti-NIMP-R14 antibody to count neutrophils that were reported as number per mm^2^ of dermis-hypodermis and SLCT layers. Doted lines indicated steady state neutrophil levels in the dermis-hypodermis layer of wt and Cxcr4^+/1013^ mice. C: Filarial load in the pleural cavity 20 days p.i. in wt (open dots) and Cxcr4^+/1013^ (black dots) mice injected with PBS (Ctrl) or with anti-Ly6G antibody clone 1A8 (left panel, Ab + Inf) or clone NIMP-R14 (right panel, Ab + Inf). Recovered worms were counted (nF). Results are expressed as mean +/- SEM, n = 4 to 8 (clone 1A8) and n = 4 to 6 (clone NIMP-R14). One-way ANOVA then Bonferroni *: p < 0.05; ***: p < 0.001; ns: not significant.

The possibility that the high number of neutrophils present in the skin of mutant mice contributed to the resistant phenotype of these mice was further investigated by depleting neutrophils prior to infection. To do so, mice neutrophils were selectively depleted by a classical method based on a single intraperitoneal injection of anti-Ly6G antibodies 6 hours before SC inoculation of filarial larvae. Either the anti-Ly6G 1A8 clone targeting more specifically neutrophils (*i*.*e*. ly6G+ cells) or the NIMP-R14 clone that can also deplete monocytes *(i*.*e*. ly6C+ cells) [51–53]. Depletion of circulating neutrophils was already effective at the time of infection (Fig 5A, D0). Then at 10 days p.i., blood neutrophil levels in wt or Cxcr4^+/1013^ mice recovered values in the same range than those of control mice (i.e. infected mice pre-injected with PBS). Importantly, depletion was also dramatic on skin-resident neutrophils as early as 6 hours after anti-Ly6G antibody injection leading to a 5 to 10 fold decrease compared with constitutive levels in wt and Cxcr4^+/1013^ mice, respectively (Fig 5B). During the time-span of the experiment, lymphocyte numbers remained unchanged between control and neutrophils-depleted wt or mutant mice. We then compared the number of worms recovered in the pleural cavity 20 days p.i. in control and neutrophils-depleted wt or mutant mice. Strikingly, neutrophil depletion with anti-Ly6G antibodies whatever the antibody used (1A8 or NIMP-R14 clones), dramatically increased the number of filariae recovered in the pleural cavity of Cxcr4^+/1013^ mice to levels similar to those reached in the wt mice (Fig 5C). In contrast, neutrophil depletion had no significant effect on the worm burden in wt mice although some increasing trend was observed upon treatment with the NIMP-R14 clone, which also affects ly6C+ monocytes (Fig 5C, right panel). These results indicated that the sole depletion of neutrophils, also affecting skin-resident ones, reverted the mutant mice to a wt phenotype, supporting a critical role for this population in the resistance of Cxcr4^+/1013^ mice to filarial infection.

### Altered neutrophil functions in Cxcr4^+/1013^ mice

**Fig 6:**
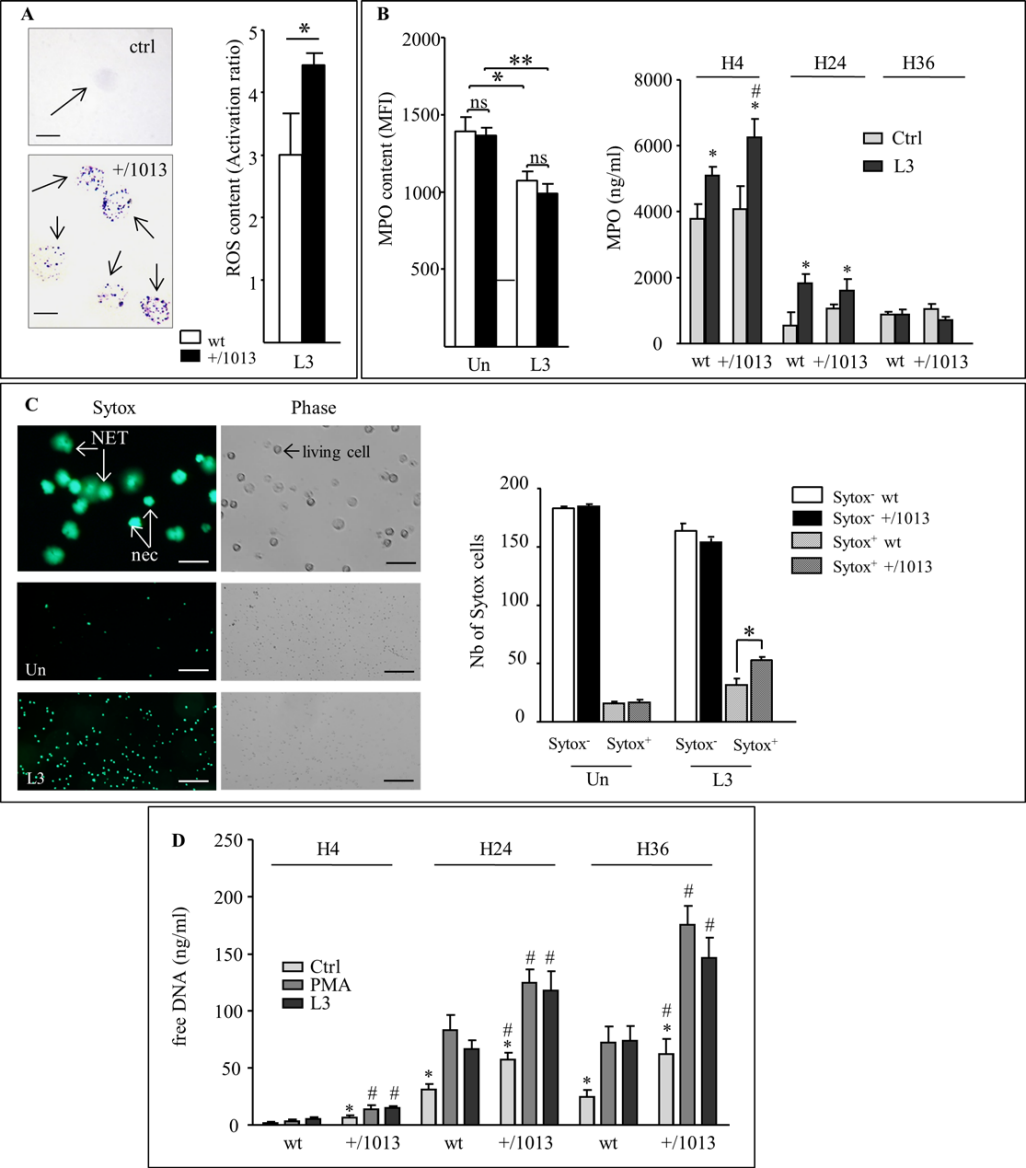
Neutrophils from Cxcr4^+/1013^ mice display higher responses to filariae exposure. A: Left panel: microscopic nitroblue tetrazolium (NBT) assay in wt and Cxcr4^+/1013^ mice. Production of superoxide anion (O2^-^) by BM-neutrophils-derived mice is revealed by the black NBT deposits (arrows) within the cells (lower panel). Scale bar = 20μm. Representative pictures of an unstained neutrophil (above) and NBT stained neutrophils (below) reporting > 90% of cells with NBT deposits. Experiment has been performed twice with wt (n = 3–4 mice) and mutant mice-derived neutrophils (n = 3–4 mice). Right panel: Relative reactive oxygen species (ROS) content measured *via* a Dichloro-dihydro-fluorescein diacetate (DCFH-DA) assay in wt (white bars) or Cxcr4^+/1013^ (black bars) mice neutrophils activated or not with L3 (20 L3 for 15 minutes). ROS content was expressed as the ratio of DCFH-DA signal of L3-actived cells relative to unstimulated cells (Activation Ratio). Experiment has been performed twice with wt (n = 5 and 4 mice) and mutant mice-derived neutrophils (n = 5 and 4 mice). T-test: *: p < 0.05. DCFH-DA signal of unstimulated cells were not statistically different (p = 0.5421) between wt (MFI = 7923 +/- 909 SEM) and Cxcr4^+/1013^ (MFI = 8811 +/- 1058 SEM) mice. B: Left panel: MPO content in wt (white bars) or Cxcr4^+/1013^ (black bars) derived neutrophils left unstimulated (Un) or after incubation with L3 (20 L3 for 1 hour). Samples were permeabilized and stained with anti-MPO-FITC antibodies and assessed by flow cytometry. Results expressed as MFI are from one representative experiment out of two (mice number: n = 5). Two-way ANOVA then Bonferroni *: p < 0.05, **: p < 0.01. Right panel: extracellular myeloperoxidase contents in culture supernatants measured using an MPO ELISA kit. Results are expressed as mean +/- SEM of two independent experiments pooled together (mice number: n = 3). Two-way ANOVA then Bonferroni. *: p < 0.05 when comparing stimulation factor (RPMI or L3), #: p < 0.05 when comparing mouse strains for a given time point and treatment. C: Quantification of necrotic and NETs releasing neutrophils left unstimulated or incubated with L3 (20 L3 for 4 hour**s)** followed by staining of the extracellular DNA with SYTOX. Left panel: The top panels illustrated the different states of neutrophil activation and were from wt neutrophils stimulated with L3. Living cells were not fluorescent and among Sytox^+^ cells, NETs-releasing neutrophils (NET, arrows) are harboring a Sytox^+^ halo that distinguished them from necrotic neutrophils (nec, arrows). Scale bar: 50μm. Other panels are representative of neutrophils of wt mice either left unstimulated (Un) or stimulated with L3. Scale bar: 500μm. Right panel: cells were analyzed (200 events) and the number of Sytox positive cells (Sytox +, stripped bars) and of Sytox negative cells (Sytox -, plain bars) for wt (white bars) or Cxcr4^+/1013^ (black bars) neutrophils were reported for each condition. Number of mice: n = 4. D: Mouse neutrophils were incubated with L3 (10 larvae) or 100 nM PMA for 4, 24 and 36 hours. Culture supernatants were analyzed for extracellular DNA using the PicoGreen assay. Results are expressed as mean +/- SEM of two independent experiments pooled together (number of mice: n = 3). Two-way ANOVA then Bonferroni. *: p < 0.05 when comparing stimulation factor (RPMI, PMA or L3), #: p < 0.05 when comparing mouse strains for a given time point and treatment.

Collectively, these data identified a critical role for skin neutrophils in the host protective mechanism against primary L3 infection. The resistant phenotype displayed by Cxcr4^+/1013^ mice might underlie, either that neutrophils must be in elevated numbers at the point of infection, and/or that mutant mice neutrophils have a heightened activation state or modified functions. From previous analyses of neutrophils derived from patients suffering from the WHIM syndrome [54] it is anticipated that Cxcr4^+/1013^neutrophils would be also prone to an enhanced Cxcl12-dependent chemotaxis [41]. Chemotaxis assays performed on BM-isolated neutrophils indeed confirmed that neutrophils derived from Cxcr4^+/1013^ mice were more sensitive to Cxcl12 than their wt counterparts (S7 Fig). This increased Cxcl12-induced chemotaxis was similarly displayed by neutrophils isolated from infected mutant mice 20 days p.i (S7 Fig). Moreover, neutrophils derived from wt and mutant mice, infected or not, were found to express equivalent levels of Cxcr4 and Cxcr2 receptors and displayed comparable chemotactic response to the Cxcr2 agonist Cxcl1 (S7 Fig). These results thus indicated that Cxcr4^+/1013^ mice derived neutrophils display *in vitro* a selective enhanced responsiveness to Cxcl12-induced chemotaxis as a consequence of the gain-of-CXCR4-function they harbor. We then investigated *in vitro* other neutrophil functions such as the capacity of these cells to produce reactive oxygen species (ROS) and to undergo NETosis. ROS production was assessed with a nitroblue tetrazolium (NBT) assay. Results indicated that both wt and mutant mice-derived neutrophils were able to reduce the colorless NBT to black deposits within the cells indicating that the production of the superoxide anion (O2-) was not altered in neutrophils derived from the Cxcr4^+/1013^ mice (Fig 6A). In contrast, quantification of the oxidative burst revealed that ROS production by neutrophils from both wt and Cxcr4^+/1013^ mice was increased upon exposure to L3 and significantly heightened in neutrophils derived from Cxcr4^+/1013^ mice as compared with wt mice ones (Fig 6A, right panel). This oxidative burst in response to L3 was associated with a significant decrease in the intracellular content of myeloperoxidase (MPO) in neutrophils from both wt and Cxcr4^+/1013^ mice (Fig 6B, left panel), which was mirrored by an increase of the neutrophil-released MPO (Fig 6B, right panel). Interestingly, neutrophils from Cxcr4^+/1013^ mice released higher levels of MPO to the medium, indicating a stronger susceptibility to the degranulation process induced by exposure to L3 (Fig 6B, right panel). Extracellular release of MPO, neutrophil elastase, histones and chromatin decorated with numerous active proteins are the signature of NETs formation. We therefore investigated the potential induction of NETosis upon neutrophils exposure to L3 larvae by quantifying both cell viability and extracellular DNA release using the cell-impermeable SYTOX dye (Fig 6C, left panel). Quantification indicated that upon 4 hours exposure with L3, neutrophils purified from Cxcr4^+/1013^ mice were significantly more engaged into a cell-death program than those from wt mice (Fig 6C, right panel). The presence of extracellular DNA in culture supernatants increased with the exposure time to L3 larvae (from 4 to 36 hours) and was more marked in cultures with Cxcr4^+/1013^ BM-derived neutrophils suggesting that these cells are more prone to release NETs (Fig 6D). Examination of immunofluorescence slides of skin from infected and control mice indeed revealed granular structures co-staining with MPO, neutrophil elastase and DAPI, which hallmark NETs structures [55], strongly supporting the existence of neutrophils undergoing NETosis in the skin of mutant mice infected with L3 larvae (S8 Fig).

## Discussion

We provide compelling evidence revealing the potential for skin neutrophils to contribute in the early host defense against primary *L*. *sigmodontis* infection by using the CXCR4-gain-of-function Cxcr4^+/1013^ neutropenic mice. These mice found to be highly resistant to primary infection allowed us to demonstrate that (i) mice harbor an elevated numbers of dermal neutrophils in steady state the depletion of which, prior to infection, ablates the resistant phenotype of the mutant mice, and that (ii) mutant mice-derived neutrophils produce increased amount of ROS mediators and NETs upon L3 larvae stimulation, as compared with wt mice-derived neutrophils. Further, an original setting of intravenous delivery resulted in a strong enhancement of the parasitic burden, which strikingly reached similar levels in both wt and mutant mice. This demonstrates that most of the incoming L3 larvae including in wt mice are destroyed in the skin upon typical SC infection thus strengthening evidence in favor of the essential role of the skin in the protective mechanism against primary L3 infection. Of note, the rate of larvae recovery in both mice upon IV delivery did not reach 100% success. One could argue that some L3 within the inoculums may be unviable, but it mainly suggests that the control of the filarial load does not only take place in the skin. Previous studies indeed revealed that among the 70% of larvae that are leaving the skin within the first day, only one third are reaching the pleural cavity [56] with some L3 being found in pulmonary alveoli and arteries [56] suggesting that the lung could act as a clearance organ [57]. Of importance and in line with this, the IV infection setting experimentally supported for the first time the Wenk’s early hypothesis that the infective larvae might pass through the cardiopulmonary blood system to reach the pleural cavity [4].

We found that mutant mice display a heightened steady state number of neutrophils in the skin. Although the mechanism of this increase remains to be determined, the enhanced expression of Cxcl12 in the dermis of mutant mice combined with the increased chemotaxis toward the chemokine displayed by the mutant mice-derived neutrophils likely contribute to this process. Numerous evidences support the hypothesis of a major contribution of this neutrophil resident pool in the protective mechanism of the mutant mice against primary L3 infection. First, the selective and transient depletion of neutrophils prior infection, which also affected neutrophils in the skin, resulted in normalization of the rate of larvae recovery in the pleural cavity of mutant mice; second, recruitment of neutrophils in the skin of wt and mutant mice 6 hours p.i. led to similar levels in both mice and was mirrored by a neutrophilia in both animals; third, infection via IV route bypassed the protective mechanism of the mutant mice and; fourth, mutant mice display normal steady state numbers of eosinophils, mast cells and macrophages in the skin, suggesting that these innate immune cells do not participate, at least quantitatively, in the resistant phenotype of the mutant mice. These results however do not rule out the potential qualitative contribution of these cells in the control of filarial infections notably in the setting up of adaptive immune response. The protective role of eosinophils was indeed described in mice vaccinated with irradiated L3 larvae [12, 13] and that of mast cells in filarial-infected CCL17 deficient mice [7]. The blood leukopenia affecting the mutant mice, which constitutes a finely tuned sensor of the leukocytosis induced by the infection, affected neutrophils and eosinophils that were found to transiently reach normal levels upon infection. Although this process is not related to the early protective mechanism of mutant mice it supports the general concept that leukocyte trafficking promoted by the infection might impact the adaptive immune response against filarial infection. Moreover it indicates the potency of filarial-antigens to induce leukocytosis as demonstrated upon injection of filarial crude extract. Such early neutrophilia (2 hours p.i) may be related to excretory-secretory (ES) proteins released by filarial nematodes and which originate from the oesophageal glands, the anterior sensory glands (amphids), the posterior sensory glands (phasmids), the secretory pore, the hypodermis through transcuticular secretion but also from exosome release [58]. Notably, we have recently reported that the excretory-secretory proteins from L3 differ quantitatively and qualitatively from the other stages of *L*. *sigmodontis* [59].

We investigated the possibility that the early protective mechanism of mutant mice might involve a higher activation of skin resident neutrophils as a consequence of the Cxcr4-gain of function. We indeed found an abnormally enhanced migration of the mutant-derived neutrophils toward CXCL12, thus extending our previous observation made in this mouse model [41]. Moreover, with regard to the large length of the L3 larvae (*i*.*e*. about 750 μm) [58, 60] we sought to investigate the potential of neutrophils in inducing NETs that can be released by in response to microbe size-sensing [61]. The NETs were reported to capture Gram-positive and Gram-negative bacteria, fungi, and viruses [62–64] as well as *Apicomplexa* parasites, *Leishmania*, *Eimeria*, *Plasmodium*, and *Toxoplasma* [65] and the *Strongyloides stercoralis* nematode [66]. NET formation or NETosis is a gradual process notably involving ROS generation, transport of MPO and the extracellular release of chromatin [67–69]. Both wt and mutant mice-derived neutrophils were able to generate ROS mediators upon L3 stimulation likely causing NETosis. Importantly, mutant mice-derived neutrophils display heightened levels of ROS content and increased release of MPO and extracellular DNA when cultured with L3 and were more prompt to death as compared to their wt counterpart. Whether the filarial excretory-secretory proteins and/or the bacterial content (*i*.*e*. *Wolbachia*) also contribute to activation of neutrophils, including triggering of NETosis in response to live L3 cannot be excluded. Further work is needed to investigate the mechanisms by which NETs contribute in L3 larval entrapment and killing, which can be indirect as suggested by recent work on the *Strongyloides stercoralis* nematode model [66].

In summary, our findings suggest that the higher responsiveness exhibited by Cxcr4^+/1013^ neutrophils may be critical in the mutant mice-protective antifilarial response thus accounting for the fact that neutrophil depletion in control wt mice does not affect parasitic burden. Hence, they emphasize the potential of skin resident neutrophils to contribute in the early host defense against filarial infections when they are sufficiently activated and present in significant numbers prior infection. Such increase of the proportion of more functionally active neutrophils maybe envisioned in a wild type host environment during inflammatory processes and the subsequent abnormal increase of aged neutrophils that represent an overly active subset with notably enhanced propensities to form NETs [70, 71] or in the context of co-infections. Indeed, the immune responses evoked by bacterial or viral infections are associated with changes in the local cytokine environment and increases in the numbers and the activation state of neutrophils that could have implications for the outcome of filarial infections in light of recent findings highlighting interplay between host immune responses against parasites and virus in the course of co-infection (reviewed in [72]). Finally, our results identifying an immuno-modulatory role for the CXCL12/CXCR4 pathway could have implications for the development of therapies that should be further studied in filarial nematodes-infected individuals.

## Materials and Methods

### Ethics statement

All experimental procedures were carried out in strict accordance with the EU Directive 2010/63/UE and the relevant national legislation, namely the French “Décret no 2013–118, 1er février 2013, Ministère de l’Agriculture, de l’Agroalimentaire et de la Forêt”. National license number 75–1415 approved animal experiments: protocols were approved by the ethical committee of the Museum National d’Histoire Naturelle (Comité Cuvier, License: 68–002) and by the “Direction départementale de la cohésion sociale et de la protection des populations” (DDCSPP) (No. C75-05-15).

### Parasites, mice, infection, treatments

The filaria *L*. *sigmodontis* were maintained in our laboratory and infective third-stage larvae (L3) were recovered by dissection of the mite vector *Ornithonyssus bacoti* [73] as previously described [74, 75].

Wt and Cxcr4^+/1013^ mice were bred in our animal facilities on a 12-hours light/dark cycle. All data were obtained from 8–12-week-old mice. Mice were genotyped by PCR on genomic DNA as previously described [41] using specific oligonucleotide primers to distinguish the mutant and the endogenous *Cxcr4* allele.

Mouse infections were carried out by SC inoculation of 40 infective L3 in 200 μL of RPMI 1640 (Eurobio, France) into the left lumbar area of mice. Only when indicated, were mouse infections carried out by an intravenous inoculation of 40 infective L3 in 50 μL of RPMI 1640 into the caudal vein. In some experiments where indicated, mice were injected with crude extracts of *L*. *sigmodontis* worms which were obtained from the homogenization and sonication of L3 recovered from infected mites; the L3 derived crude extract concentration was determined by a Bradford assay (Pierce) following the manufacturer’s instructions. After centrifugation, the supernatant was collected and the protein content was determined by the modified Bradford method (BCA Protein Assay kit, Pierce). Mice then received 10 μg of crude extract either subcutaneously in 200 μL RPMI 1640 or intravenously in 50 μL of RPMI 1640 as above. Mice were sacrificed at 6 hours, 20 and 80 days post-inoculation (p.i.) as indicated below.

### Blood leukocyte counts

Blood smears and blood cell counts were performed before and throughout the challenge at different time points. Blood smears were obtained from the tail vein, stained with May-Grünwald-Giemsa (VWR, France) and the percentages of the different leukocyte populations were determined for 200 cells. Total blood cell counts were determined from tail blood mixed with 1% acetic solution (1:5 vol) using a hemocytometer (KOVA Glasstic Slide).

### Filarial load, pleural leukocyte recovery, flow cytometry

At the indicated time p.i., mice were anaesthetized and sacrificed by terminal bleeding. Blood was allowed to clot for 30 min at room temperature then centrifuged and sera were collected and kept at -20°C until further use. The pleural cavity was washed with 10 mL of cold phosphate-buffered saline (PBS, EUROBIO, France), as previously described [12]. The infiltrating cells and the worms were collected from the pleural wash for further analysis. Pleural washes were then frozen at -20°C until further use. Worms were fixed *in toto* with 4% formaldehyde in cold PBS to avoid body shrinkage and the gender and development stages of the worms were analyzed by light microscopy. PleCs were centrifuged at 250 g for 8 min at 4°C, resuspended in 2 ml RPMI supplemented with 2% foetal calf serum (FCS, EUROBIO, France) and then counted in PBS/ 0.04% trypan blue (Sigma-Aldrich) using a haemocytometer (KOVA Glasstic Slide). Proportions of the different leukocyte populations were determined by flow cytometry using the following rat anti-mouse antibodies: anti-F4/80-APC (clone BM8), anti-SiglecF-PE (clone E50-2440), anti-Ly6G-FITC (clone RB6-8C5), anti-CD3-PE (clone 145-2C11), and anti B220-FITC (clone RA3-6B2). All antibodies were purchased from eBioscience except the anti-SiglecF-PE (BD Pharmingen) and used at a 1/40 dilution. Flow cytometry analysis was performed using a FACSVerse flow cytometer running the FACSuite software (BD Biosciences). Acquisition and analyses were performed as described in S2 Fig.

### Immunohistological analyses

Two days prior to the challenge, mice were anesthetised and depilated by means of Veet hair removal cream on an area of flank skin in the left inguinal region over the inguinal lymph node. For the challenge, wt and Cxcr4^+/1013^ mice were then inoculated either with 40 infective larvae or RPMI as a control and sacrificed 6 hours p.i. Skin sections of 1 cm^2^ were taken from the inoculation site, fixed overnight in 4% paraformaldehyde (PFA, VWR, France) and embedded in paraffin. Paraffin sections (5 μm) were stained with hematoxylin-eosin (H&E, VWR, France) or toluidine blue (VWR, France) for the detection of eosinophils or mast cells respectively. A modified Hematoxylin Eosin (HE) staining, including an alkaline eosin solution-staining step (pH 8.4) for 20 seconds has been selectively chosen to minimize background tissue eosin staining. For immunohistochemistry analyses, i.e. macrophages and neutrophils visualization, deparaffinized slides were incubated with the anti-F4/80 (clone CI:A3-1, Abdbiotech) or anti-NIMP-R14 (clone NIMP-R14, Abcam) antibodies respectively in 3% PBS-BSA overnight at 4°C. A peroxidase-based system was used for detection. For immunofluorescence staining, i.e. NETs, deparaffinized slides were incubated with anti-MPO (3.3 μg/mL, AF3667 R&D, Lille, France) or anti-Elastase (ELA) (5 μg/mL, Ab68672, Abcam, Paris, France) antibodies followed by staining with goat anti-rabbit Alexa Fluor 488 or anti-rat Alexa Fluor 594 (20 μg/mL, A-11034 and A-11007, respectively, Life Technologies, Saint-Aubin, France). DNA was visualized upon DAPI counterstaining. Images were acquired using the digital slide scanner HPF-NanoZoomer RS2.0 (Hamamatsu) coupled to a high definition 3-CCD digital camera. In some cases, images were acquired using an Olympus DP72 camera coupled to an Olympus BX63 motorized microscope running the cellSens Dimension (v 1.9) software.

### Neutrophil depletion

Neutrophils were depleted by a single intra-peritoneal injection of either 0.25 mg of anti-Ly6G clone NIMP-R14 (AdipoGen) or 0.5 mg of anti-Ly6G clone 1A8 (BioXCell) 6 hours prior to infection (performed as described above in the Material and Methods Section 2). The two antibodies have been used extensively to deplete neutrophils in mice [51–53] with a potentially better selectivity for neutrophils of the 1A8 clone with regard to other Gr-1^+^ cells [51]. The effect of the treatment was assessed on blood and skin-resident neutrophils. Circulating neutrophil depletion was confirmed by blood smears and differential blood cell counts from 6 hours to 20 days post-injection of the depleting antibody. The effect of the depletion on skin-resident neutrophils was assessed by immunohistological analyses (see above, Material and Methods section 5) using the anti-NIMP-R14 antibody on paraffin-embedded skin sections of control or injected mice 6 and 12 hours post-injection of the depleting antibody.

### Functional assays

Chemotaxis assays were performed on BM leukocytes using a Transwell assay as previously described [34]. Leukocytes were isolated from the BM of wt and Cxcr4^+/1013^ control or infected (at 20 days p.i.) mice and resuspended in assay buffer (HBSS medium supplemented with 20mM HEPES and 0.5% bovine serum albumin). Leukocytes (3 x 10^6^ cells) were then added to the upper chamber of transwell filters (Millipore, 3μm pore diameter) that were placed in 24-well cell culture plates containing 300μl assay buffer with or without the indicated chemokines. In some experiments cells were pre-incubated at 37°C with Cxcl12 10 μM and AMD3100 200 μM for 30 min before being placed in the upper transwell chamber to confirm the specificity of the Cxcl12-dependent migration upon Cxcr4. Chambers were then incubated for 60 min at 37°C with 5% CO_2_ and the cells that migrated to the bottom chamber were recovered and stained with anti-ly6G-FITC antibody for flow cytometry analysis. The number of neutrophils that migrated into the bottom chamber was determined by a flow cytometer (BD Biosciences) with relative cell counts obtained by acquiring events for a set time period of 30s. Chemotactic indexes were then calculated by dividing the number of neutrophils that were counted in the chemokine stimulated well by the number of neutrophil that were obtained in the non-stimulated well.

Cell surface expression of Cxcr2 and Cxcr4 was also assessed on BM leukocytes from naïve and infected mice at 20 d.p.i. Neutrophils were identified with an anti-ly6G-FITC antibody and stained with either anti-Cxcr2-APC (clone 242216, R&D) and anti-Cxcr4-PE (clone 247506, R&D) antibodies or their respective control isotypes. Samples were processed by a FACSVerse flow cytometer (BD Biosciences) and analyzed using FACS Suite software. BM leukocytes from naive wt and Cxcr4^+/1013^ control mice were subjected to a discontinuous 72–64% Percoll density gradient centrifugation in 15 mL Falcon tubes for 30 minutes at 4°C. Mature neutrophils were collected at the 72–64% interface (purity > 93%), washed three times in cold PBS then resuspended in PBS at the working concentration of 10^6^ cells/mL. The following assays were performed: The production of superoxide anions (O2^-^) was investigated using a microscopic NBT assay. In brief, 10^5^ mature neutrophils per mouse were incubated in an 8-well Lab-Tek Chamber Slide for 1 hour at 37°C allowing the cells to attach to the plate. The supernatant was then discarded and 100 μL of NBT (1 mg/mL) was added to each well. Cells were incubated for 1 hour at 37°C and slides were analyzed by optical microscopy (Olympus BX63 microscope). The slide was counterstained 3 min with a 10% Giemsa solution then water washed. Black NBT deposits within the cells revealed the production of O2^-^. ROS content was measured *via* a dichloro-dihydro-fluorescin diacetate (DCFH-DA) assay (Sigma-Aldrich). Briefly, 10^5^ mature neutrophils were incubated with 125 μM DCFH-DA in a 96-well cell culture plate for 15 minutes at 37°C to allow its entry into cells where it get converted in DCFH. Cells were then left unstimulated or stimulated with 20 L3 for 15 minutes at 37°C. Neutrophil oxidative response changes DCFH to green fluorescent DCF. The reaction was stopped by placing cells at 4°C. L3 were removed from the wells and cells were stained with anti-Ly6G-PECy7 antibody (eBioscience, clone RB6-8C5, dilution 1/40). Samples were processed by a flow cytometer (BD Biosciences) and analyzed using FACS Suite software. ROS content was expressed as an activation ratio by dividing the FITC mean fluorescence intensity (MFI) of neutrophils from L3 stimulated conditions by that from unstimulated cells.

MPO content was measured in wt or Cxcr4^+/1013^ mature mouse neutrophils (10^5^ cells) either unstimulated or stimulated with 20 L3 in a 96-well cell culture plate at 37°C for 1 hour. Cells were then permeabilized with Saponin-PBS (0.2% BSA + 0.05% saponin in PBS) and stained with anti-Ly6G-PECy7 (eBioscience, clone RB6-8C5, dilution 1/40) and anti-MPO-FITC (Hycult Biotech, clone 8F4, dilution 1/40). Samples were processed by a flow cytometer and analyzed using FACS Suite software. MPO content was expressed as MFI.

Neutrophil necrosis and NETosis were quantified by immunofluorescence light microscopy in wt or Cxcr4^+/1013^ mature mouse neutrophils (10^5^ cells) either left unstimulated or stimulated with 20 L3 in 24-well culture plates at 37°C for 4 hour. SYTOX-Green (Thermo Fisher Scientific), which does not enter into live cells, was then added (dilution 1:15000) for the detection of either extracellular DNA indicative of NETs release or intracellular DNA of necrotic neutrophils. Viable cells appear as non-fluorescent cells whereas both necrotic and NETs releasing neutrophils become fluorescent. Necrotic neutrophils are characterized by compromised membrane integrity and pluri-lobed nuclei whereas NETs releasing neutrophils display a halo (DNA area > 500 μm^2^) corresponding to chromatin decondensation and NETs release [60, 76]. Images were acquired using an Olympus DP72 camera coupled to an Olympus BX63 motorized microscope running the cellSens Dimension (v 1.9) software. Cells were counted and the numbers of necrotic, NETs releasing and living cells were determined.

Extracellular DNA was also quantified: mouse neutrophils (1x10^5^) were cultured with 10 infective larvae or 100 nM phorbolmyristate acetate (PMA) for 4, 24 and 36 h in vitro. The culture supernatants were collected and the extracellular DNA was quantified using the Quant-iT PicoGreen dsDNA Assay kit (Life Technologies), following manufacturer instructions. Samples were cultured with the PicoGreen reagent (1:1 dilution) for 5 min. The samples were measured with a spectrofluorometer at 480 nm excitation and 520 nm emission. A DNA standard curve was used to determine the concentration of free DNA from samples

### Statistical analyses

Statistical analysis was carried out using GraphPad Prism v5. Sample size, normality (Shapiro-Wilk test) and homoscedasticity (Bartlett’s test) were tested prior to further analysis. Data from separate experiments were pooled where possible. Most of the time one-way ANOVA analyses followed by a Bonferroni post-hoc test were performed. T-tests were used when comparing filarial load and lymphocytes, eosinophils and neutrophils cell counts at 20 days p.i. between wt and Cxcr4^+/1013^ infected mice. One-way ANOVAs with repeated measures were used to compare neutrophils cell counts in the blood of infected mice during the course of the infection. Significance was defined as *: p < 0.05; **: p < 0.01 and ***: p < 0.001.

## Supporting Information

S1 Fig A model of *L*. *sigmodontis* filarial infection in mice: Time course and invasion routes. Recovery rate in pleural cavity (% of inoculum, in green) and total PleCs (X106 cells, in blue) in resistant and susceptible mice in the course of the filarial infection adapted from [4, 39, 47, 77–79]. Hypothetical location and migration path of the parasite upon its inoculation in the skin of its murine host are indicated at the top and on the left side of the figure. We chose 20 days p.i. (framed in red) as a reference time point in our study based on the fact that both the filarial load and the total number of PleCs are still high in mice.
(TIF)

S2 Fig Parasitic success is dramatically reduced in Cxcr4^+/1013^ mutant mice as early as day 8 post infection. *L*. *sigmodontis* (40 larvae) were subcutaneously injected into wt and Cxcr4^+/1013^ C57BL/6 mice. Worms were harvested in the pleural cavity of the mice 8 days p.i. and counted (nF). Results are expressed as mean +/- SEM, n = 5 mice per group, MW-test *: p < 0.05.
(TIF)

S3 Fig Total number of PleCs and their composition. A: Cells were harvested and counted at necropsy at 20 days p.i. upon flushing the pleural cavity of non-infected (wt ctrl and +/1013 ctrl) and infected (wt inf and +/1013 inf) mice. Cells were then stained with F4/80-APC, Siglec-F-PE, Ly6G-FITC, CD3-PE and B220-FITC directed antibodies. B: Gating strategy: macrophages were gated as F4/80^high^ SiglecF^-^ and F4/80^int^ SiglecF^-^ cells, eosinophils as F4/80^int^ SiglecF^+^ cells, neutrophils as Ly6G^+^ cells, B lymphocytes as B220^+^ cells and T lymphocytes as CD3^+^ cells. C: Results were expressed as Box-and-Whisker plots, n = 20 for the infected mice (pool of 4 independent experiments with 5 mice per group), n = 8 for the control mice (pool of 2 independent experiments with 4 mice per group). One-way ANOVA then Bonferroni: statistical differences are illustrated by the letters a and b (panels A and C).
(TIF)

S4 Fig Steady state levels and recruitment of cells in the skin upon filariae inoculation. Skin sections were collected before inoculation (control mice, Ctrl) or excised from the inoculation site 6 hours p.i. (Infected mice, Inf), embedded in paraffin and stained with H&E for the visualization of eosinophils (A), anti-F4/80 for macrophages (B) or toluidine blue for mast cells (C). A left: Representative H&E stained skin section from wt control mice showing the different skin layers, i.e. the epidermis (e), the dermis (d), the hypodermis (h), the panniculus carnosus muscle (m) and the subcutaneous loose connective tissue layer (s). A right: Detection of eosinophils in H&E stained skin sections. Eosinophils appear as polynuclear cells with purple cytoplasm (arrows). Scale bars: 80μm (top) and 20μm (bottom). Total numbers of eosinophils per mm^2^ of skin in the dermis-hypodermis and the SLCT layer were reported in the graph. n = 7 to 8 per group. One-way ANOVA then Bonferroni *: p < 0.05, **: p < 0.01. B and C: representative sections for each group (wt and Cxcr4^+/1013^ control (Ctrl) and infected mice (Inf)) (left panels) and the total number of each targeted cell population per mm^2^ skin in the dermis-hypodermis and the SLCT layers (graphs, right panels). Representative sections: scale bar = 100μm, magnification x40. Representative x100 magnifications: scale bar = 40μm. n = 7 to 8 per group.
(TIF)

S5 Fig Cxcl12 levels in dermal stroma of wt and mutant Cxcr4^+/1013^ mice at steady state and 6 hours following infection. Images captured from scanned skin sections from mutant mice and their wt littermate that were collected from the inoculation site 6 hours after infection (Infected H6 p.i.) or injection of RPMI media (untreated). Skin sections were embedded in paraffin and stained with primary antibody for Cxcl12 (10 μg/mL, K15C clone, MABC184, EMD Millipore, Saint-Quentin-en-Yvelines, France). Bound antibody was detected using the LSAB+/HRP kit (K0679, Dako, Les Ulis, France). Sections were counterstained with hematoxylin. Arrows (in the dermis/hypodermis) and stars (in the epidermis) indicated Cxcl12 staining. Scale bars = 100 μm.
(TIF)

S6 Fig Blood neutrophils number in the 6 hours following infection. Total numbers of neutrophils (millions of cells per mL of blood) before (H0) and after SC infection (2 and 6 hours, H2 and H6) with 40 infective larvae of wt (white bars) and Cxcr4^+/1013^ (black bars) mice. Results are expressed as mean +/- SEM. T-tests *: p < 0.05, ns = not significant.
(TIF)

S7 Fig Cxcr4^+/1013^ neutrophils exhibit a gain of Cxcr4-dependent chemotaxis. A: Chemotaxis of murine BM-derived neutrophils from wt and Cxcr4^+/1013^ control mice and their infected counterparts (at 20 days p.i) in response to Cxcl1 (50nM), Cxcl12 (10nM) or Cxcl12 (10nM) + AMD3100 (200μM). Migrating neutrophils recovered in the lower chamber were gated as Ly6G-FITC^+^ cells. Results are expressed as median of n = 5–6 mice per group. One-way ANOVA then Bonferroni *: p < 0,05. B: Expression of Cxcr2 and Cxcr4 at the membrane of neutrophils isolated from the BM of wt and Cxcr4^+/1013^ control and infected mice (at 20 days p.i.). Results are expressed as median of the receptor geometric mean fluorescence intensity (MFI) n = 5 mice per group.
(TIF)

S8 Fig Visualization of NET-like structures in the skin of mutant mice 6 hours following infection. Images captured from scanned skin sections from mutant mice (+/1013) and their normal littermate (wt) that were collected from the inoculation site 6 hours after infection (Inf) or injection of RPMI media (Ctrl). Two skin sections are shown (A and B) for each condition and are from one of two independent experiments (n = 3–4 mice per group in each condition). Skin sections were embedded in paraffin and stained with primary antibody for MPO (green) or Elastase (ELA) (red) followed by staining with goat anti-rabbit Alexa Fluor 488 or anti-rat Alexa Fluor 594, respectively. DNA was visualized upon DAPI counterstaining (blue). Surrounded squares in B showing NET-like structures with merge staining for DNA, ELA and MPO are enlarged in insets (size ≈ 20–30 μm).
(TIF)

S1 Table Blood number of neutrophils the first 5 days following infection. Total numbers of blood neutrophils (millions of cells per mL of blood) measured every day the first 5 days following SC injection of 40 infective larvae in wt and Cxcr4^+/1013^ mice (n = 5). Means were compared at each time point for each mice, t-tests, ***: p < 0.001.
(TIF)

S2 Table Blood number of neutrophils throughout the course of filarial infection (Days 1–40) following IV or SC challenges. Raw blood neutrophils counts from the experiments presented in Fig 2 panel C. Neutrophil counts (millions of cells per mL of blood) were obtained from mice either IV inoculated with 40 infective larvae (IV L3 wt and IV L3 +/1013, n = 5, panel A) or 10μg L3-derived whole body extracts (IV WBE wt and IV WBE +/1013, n = 5, panel B) or SC inoculated with 40 infective larvae (SC L3 wt and SC L3 +/1013, n = 8, panel C) or 10μg L3-derived whole body extracts (SC WBE wt and SC WBE +/1013, n = 4, panel D).
(TIF)
