# Supplementary figures and images for: Neutropenic Mice Provide Insight into the Role of Skin-Infiltrating Neutrophils in the Host Protective Immunity against Filarial Infective Larvae
Source: PLoS Negl Trop Dis. 2016 Apr 25;10(4):e0004605. doi: 10.1371/journal.pntd.0004605 (PMC4844152; doi:10.1371/journal.pntd.0004605)

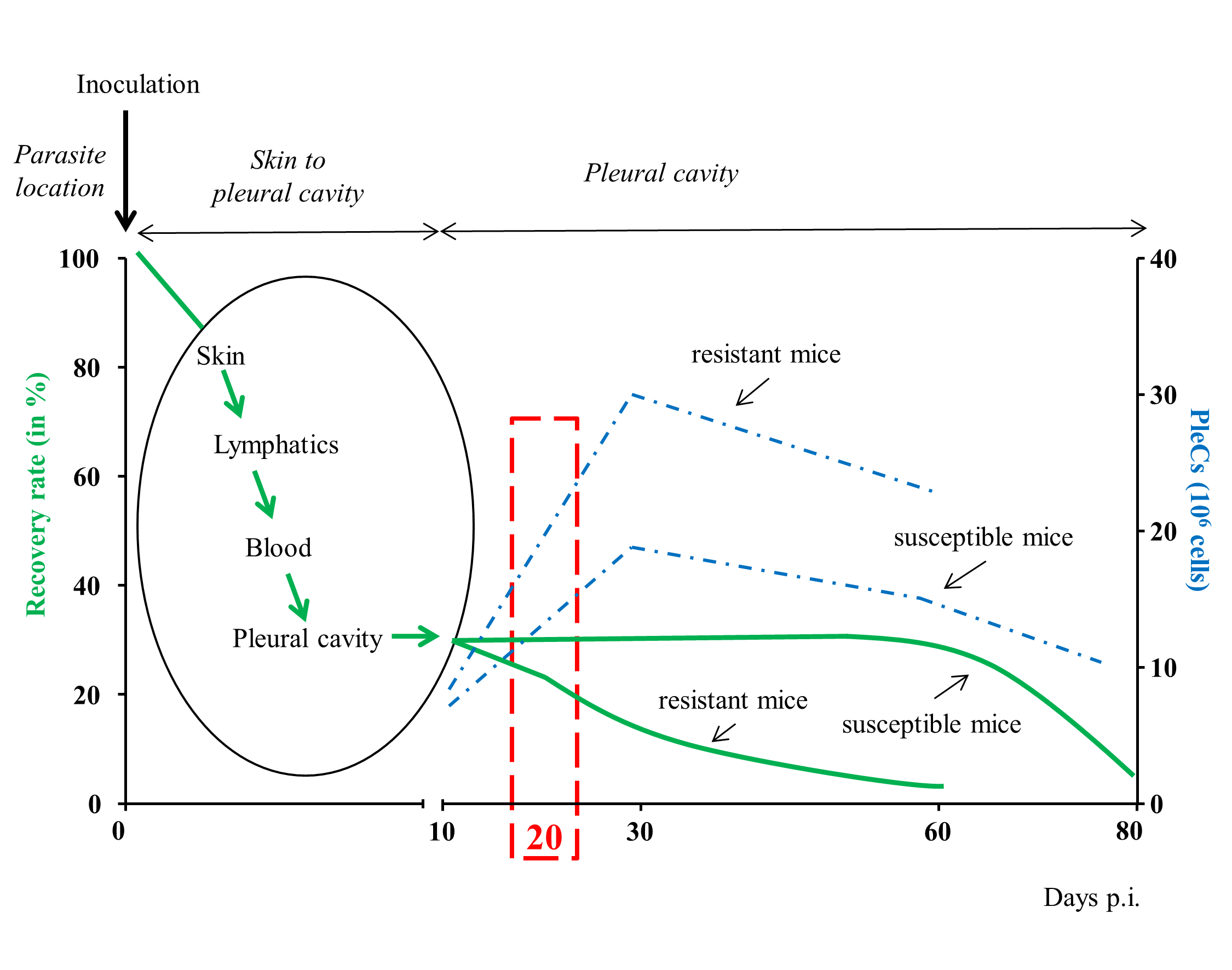

Supplement: S1 Fig — Recovery rate in pleural cavity (% of inoculum, in green) and total PleCs (X106 cells, in blue) in resistant and susceptible mice in the course of the filarial infection adapted from [4, 39, 47, 77–79]. Hypothetical location and migration path of the parasite upon its inoculation in the skin of its murine host are indicated at the top and on the left side of the figure. We chose 20 days p.i. (framed in red) as a reference time point in our study based on the fact that both the filarial load and the total number of PleCs are still high in mice. (TIF) [file pntd.0004605.s001.tif]

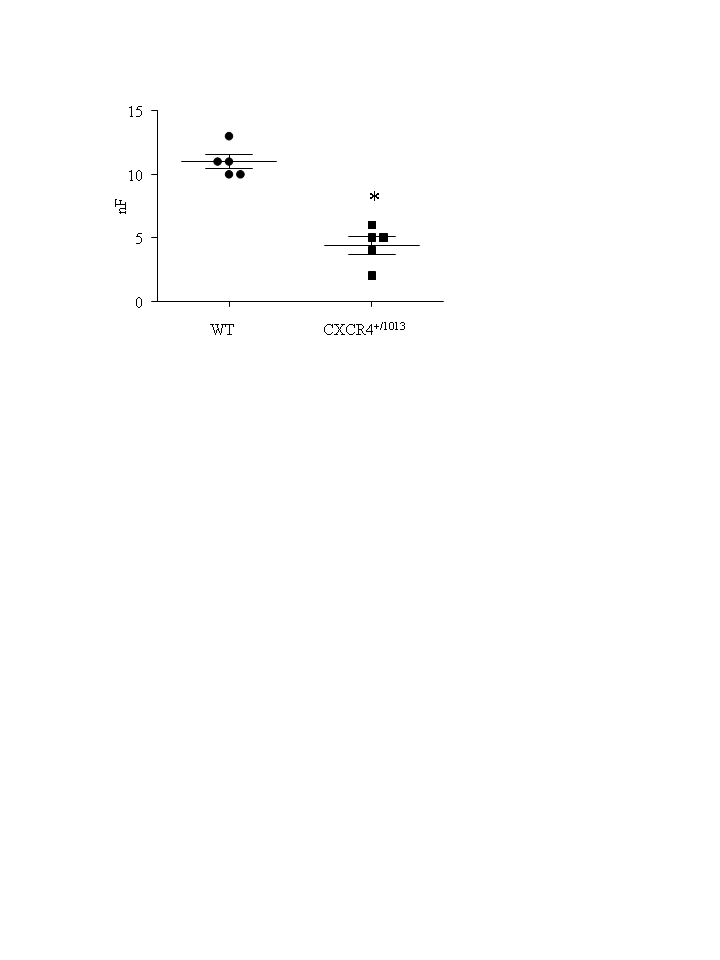

Supplement: S2 Fig — L. sigmodontis (40 larvae) were subcutaneously injected into wt and Cxcr4+/1013 C57BL/6 mice. Worms were harvested in the pleural cavity of the mice 8 days p.i. and counted (nF). Results are expressed as mean +/- SEM, n = 5 mice per group, MW-test *: p < 0.05. (TIF) [file pntd.0004605.s002.tif]

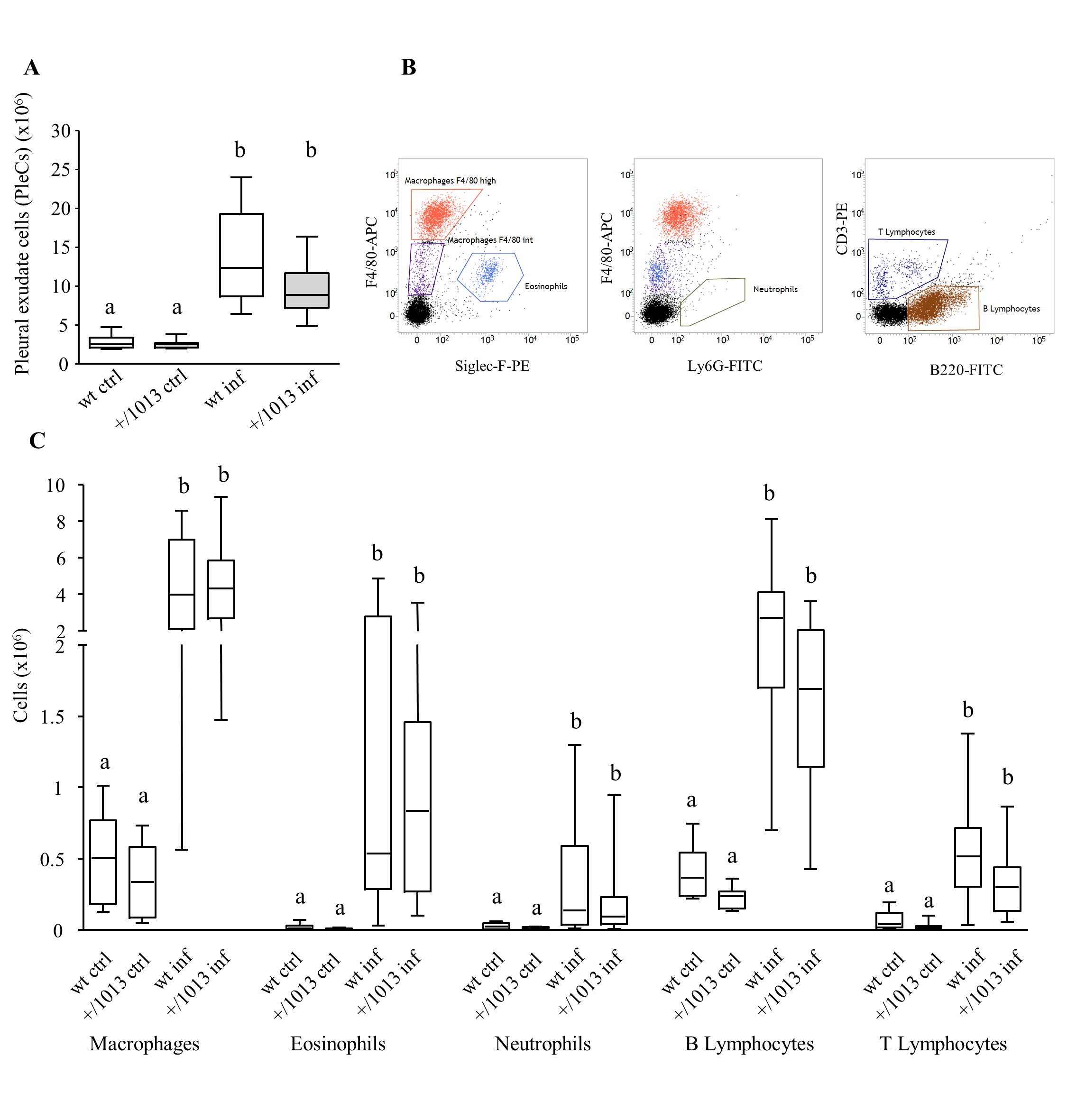

Supplement: S3 Fig — A: Cells were harvested and counted at necropsy at 20 days p.i. upon flushing the pleural cavity of non-infected (wt ctrl and +/1013 ctrl) and infected (wt inf and +/1013 inf) mice. Cells were then stained with F4/80-APC, Siglec-F-PE, Ly6G-FITC, CD3-PE and B220-FITC directed antibodies. B: Gating strategy: macrophages were gated as F4/80high SiglecF- and F4/80int SiglecF- cells, eosinophils as F4/80int SiglecF+ cells, neutrophils as Ly6G+ cells, B lymphocytes as B220+ cells and T lymphocytes as CD3+ cells. C: Results were expressed as Box-and-Whisker plots, n = 20 for the infected mice (pool of 4 independent experiments with 5 mice per group), n = 8 for the control mice (pool of 2 independent experiments with 4 mice per group). One-way ANOVA then Bonferroni: statistical differences are illustrated by the letters a and b (panels A and C). (TIF) [file pntd.0004605.s003.tif]

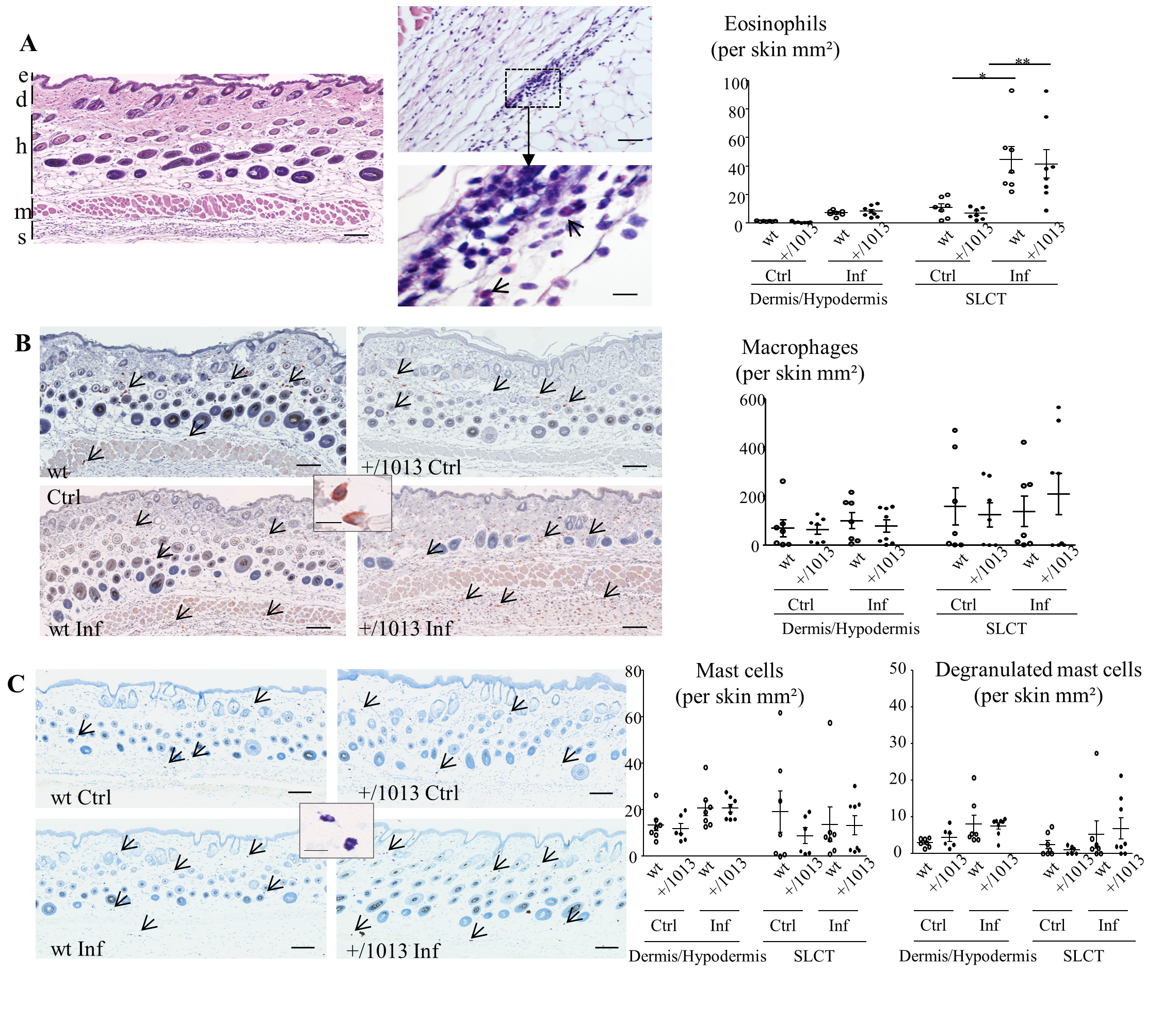

Supplement: S4 Fig — Skin sections were collected before inoculation (control mice, Ctrl) or excised from the inoculation site 6 hours p.i. (Infected mice, Inf), embedded in paraffin and stained with H&E for the visualization of eosinophils (A), anti-F4/80 for macrophages (B) or toluidine blue for mast cells (C). A left: Representative H&E stained skin section from wt control mice showing the different skin layers, i.e. the epidermis (e), the dermis (d), the hypodermis (h), the panniculus carnosus muscle (m) and the subcutaneous loose connective tissue layer (s). A right: Detection of eosinophils in H&E stained skin sections. Eosinophils appear as polynuclear cells with purple cytoplasm (arrows). Scale bars: 80μm (top) and 20μm (bottom). Total numbers of eosinophils per mm2 of skin in the dermis-hypodermis and the SLCT layer were reported in the graph. n = 7 to 8 per group. One-way ANOVA then Bonferroni *: p < 0.05, **: p < 0.01. B and C: representative sections for each group (wt and Cxcr4+/1013 control (Ctrl) and infected mice (Inf)) (left panels) and the total number of each targeted cell population per mm2 skin in the dermis-hypodermis and the SLCT layers (graphs, right panels). Representative sections: scale bar = 100μm, magnification x40. Representative x100 magnifications: scale bar = 40μm. n = 7 to 8 per group. (TIF) [file pntd.0004605.s004.tif]

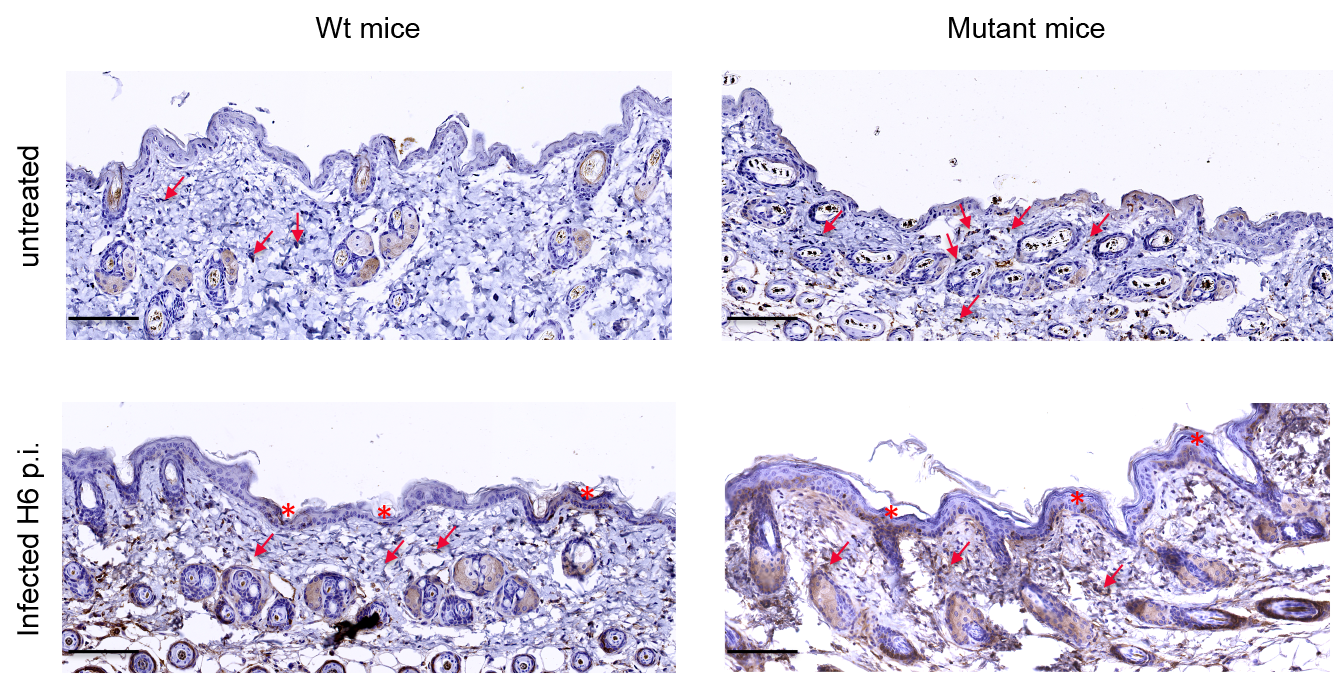

Supplement: S5 Fig — Images captured from scanned skin sections from mutant mice and their wt littermate that were collected from the inoculation site 6 hours after infection (Infected H6 p.i.) or injection of RPMI media (untreated). Skin sections were embedded in paraffin and stained with primary antibody for Cxcl12 (10 μg/mL, K15C clone, MABC184, EMD Millipore, Saint-Quentin-en-Yvelines, France). Bound antibody was detected using the LSAB+/HRP kit (K0679, Dako, Les Ulis, France). Sections were counterstained with hematoxylin. Arrows (in the dermis/hypodermis) and stars (in the epidermis) indicated Cxcl12 staining. Scale bars = 100 μm. (TIF) [file pntd.0004605.s005.tif]

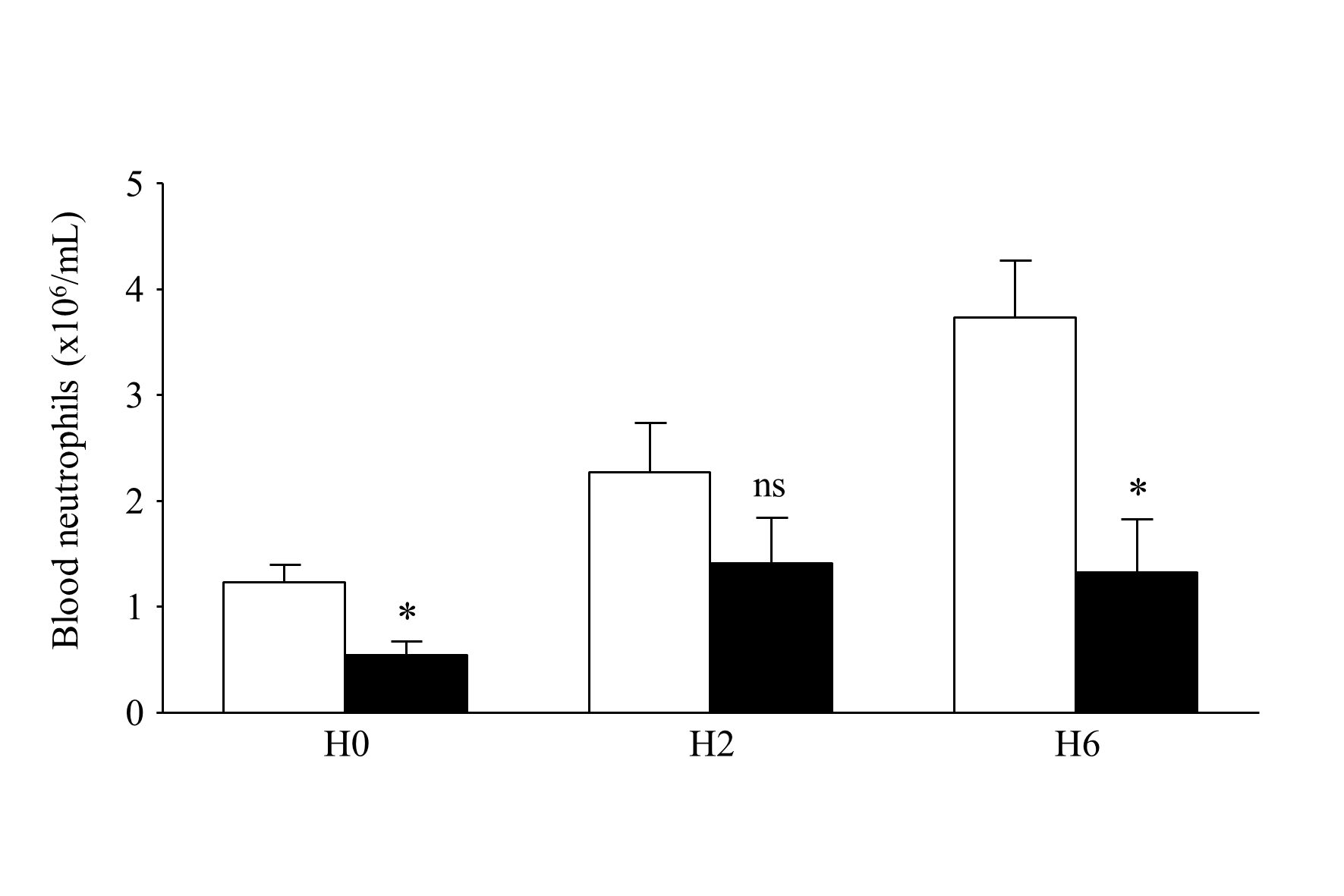

Supplement: S6 Fig — Total numbers of neutrophils (millions of cells per mL of blood) before (H0) and after SC infection (2 and 6 hours, H2 and H6) with 40 infective larvae of wt (white bars) and Cxcr4+/1013 (black bars) mice. Results are expressed as mean +/- SEM. T-tests *: p < 0.05, ns = not significant. (TIF) [file pntd.0004605.s006.tif]

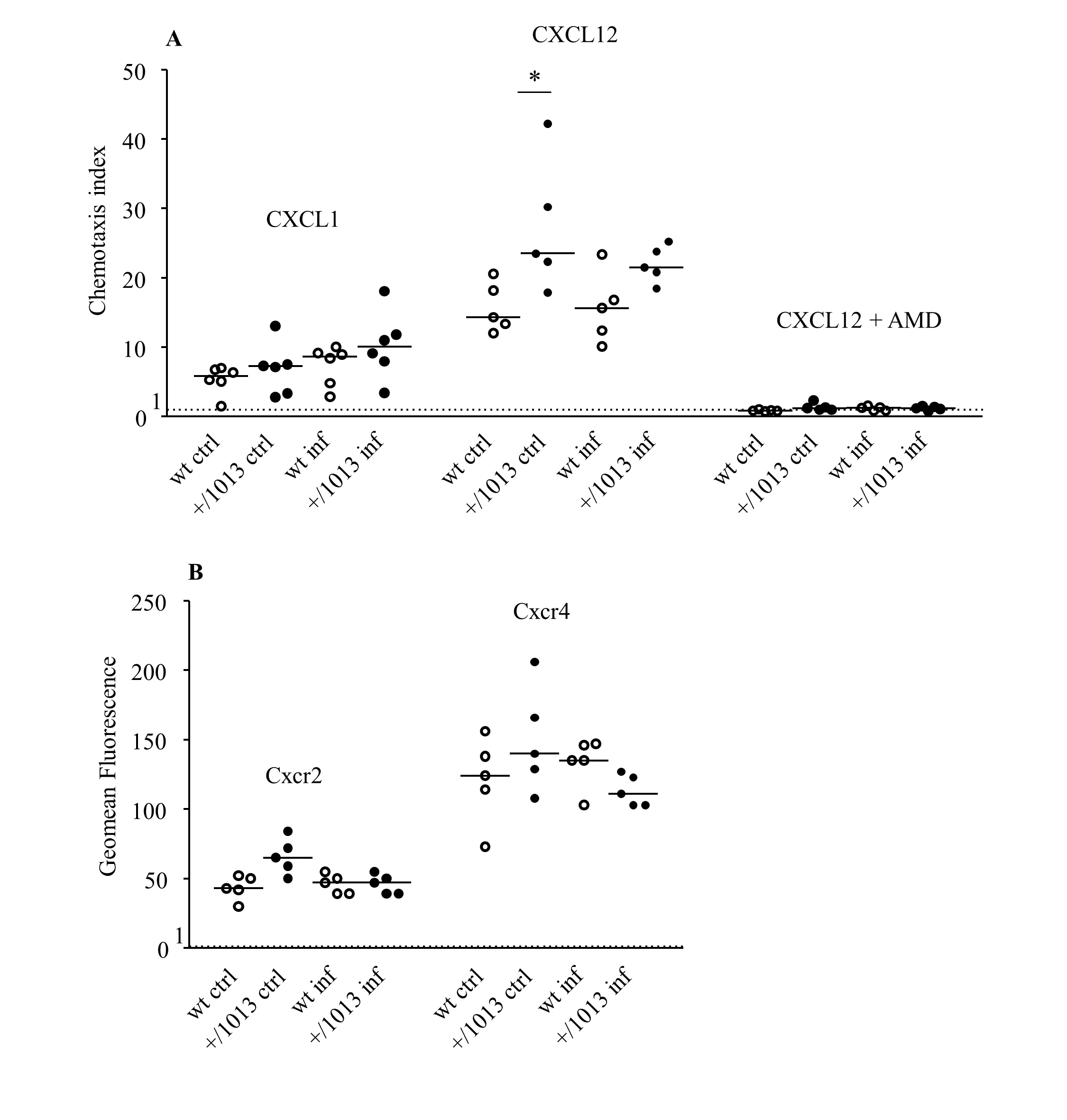

Supplement: S7 Fig — A: Chemotaxis of murine BM-derived neutrophils from wt and Cxcr4+/1013 control mice and their infected counterparts (at 20 days p.i) in response to Cxcl1 (50nM), Cxcl12 (10nM) or Cxcl12 (10nM) + AMD3100 (200μM). Migrating neutrophils recovered in the lower chamber were gated as Ly6G-FITC+ cells. Results are expressed as median of n = 5–6 mice per group. One-way ANOVA then Bonferroni *: p < 0,05. B: Expression of Cxcr2 and Cxcr4 at the membrane of neutrophils isolated from the BM of wt and Cxcr4+/1013 control and infected mice (at 20 days p.i.). Results are expressed as median of the receptor geometric mean fluorescence intensity (MFI) n = 5 mice per group. (TIF) [file pntd.0004605.s007.tif]

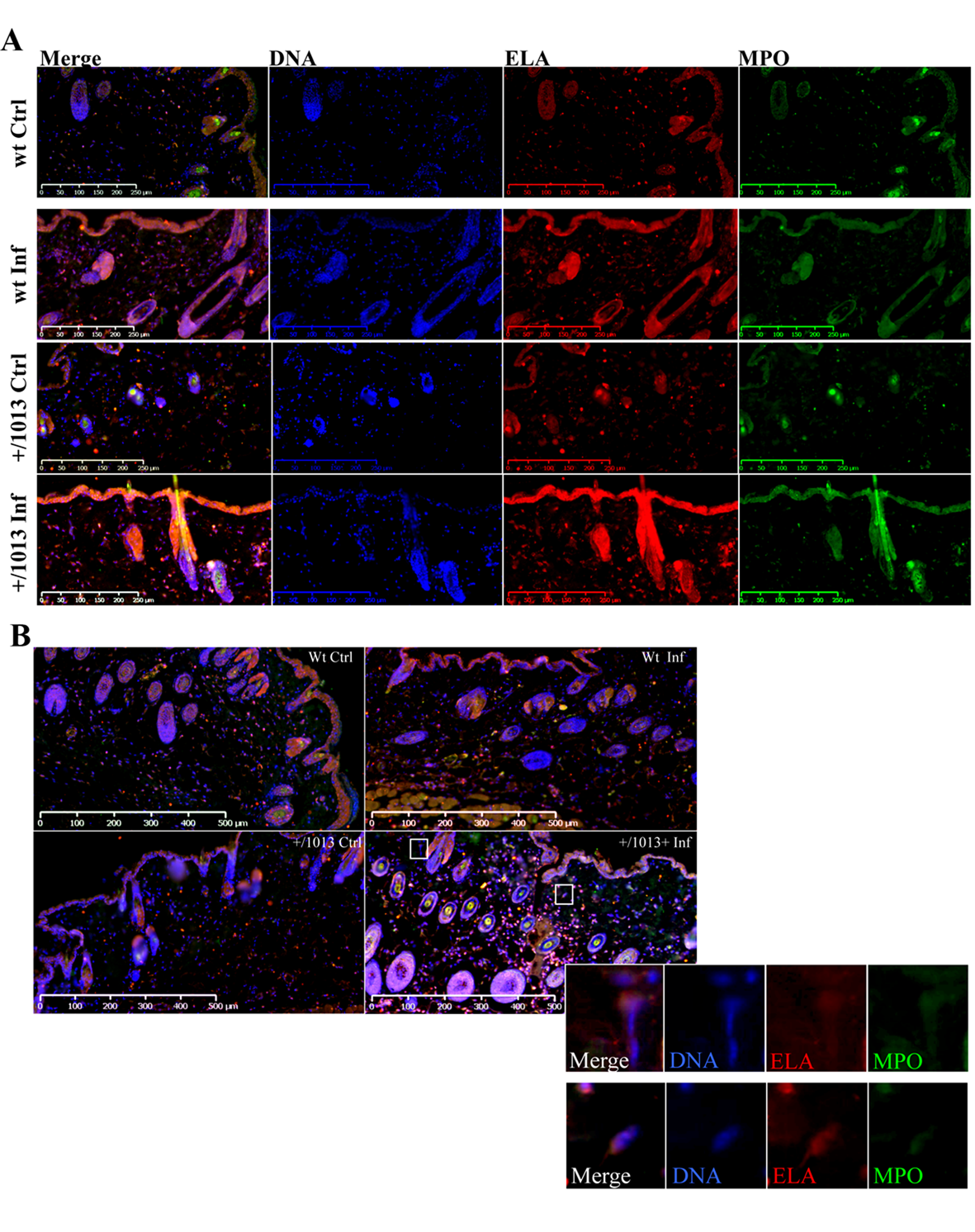

Supplement: S8 Fig — Images captured from scanned skin sections from mutant mice (+/1013) and their normal littermate (wt) that were collected from the inoculation site 6 hours after infection (Inf) or injection of RPMI media (Ctrl). Two skin sections are shown (A and B) for each condition and are from one of two independent experiments (n = 3–4 mice per group in each condition). Skin sections were embedded in paraffin and stained with primary antibody for MPO (green) or Elastase (ELA) (red) followed by staining with goat anti-rabbit Alexa Fluor 488 or anti-rat Alexa Fluor 594, respectively. DNA was visualized upon DAPI counterstaining (blue). Surrounded squares in B showing NET-like structures with merge staining for DNA, ELA and MPO are enlarged in insets (size ≈ 20–30 μm). (TIF) [file pntd.0004605.s008.tif]

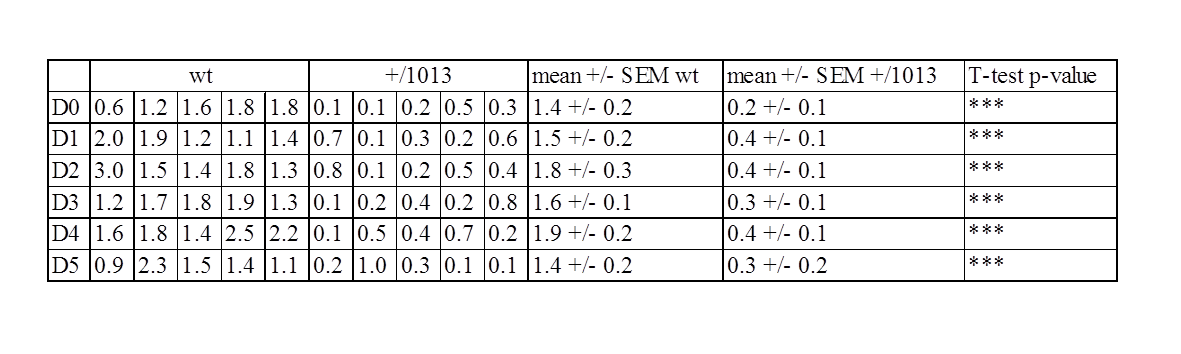

Supplement: S1 Table — Total numbers of blood neutrophils (millions of cells per mL of blood) measured every day the first 5 days following SC injection of 40 infective larvae in wt and Cxcr4+/1013 mice (n = 5). Means were compared at each time point for each mice, t-tests, ***: p < 0.001. (TIF) [file pntd.0004605.s009.tif]

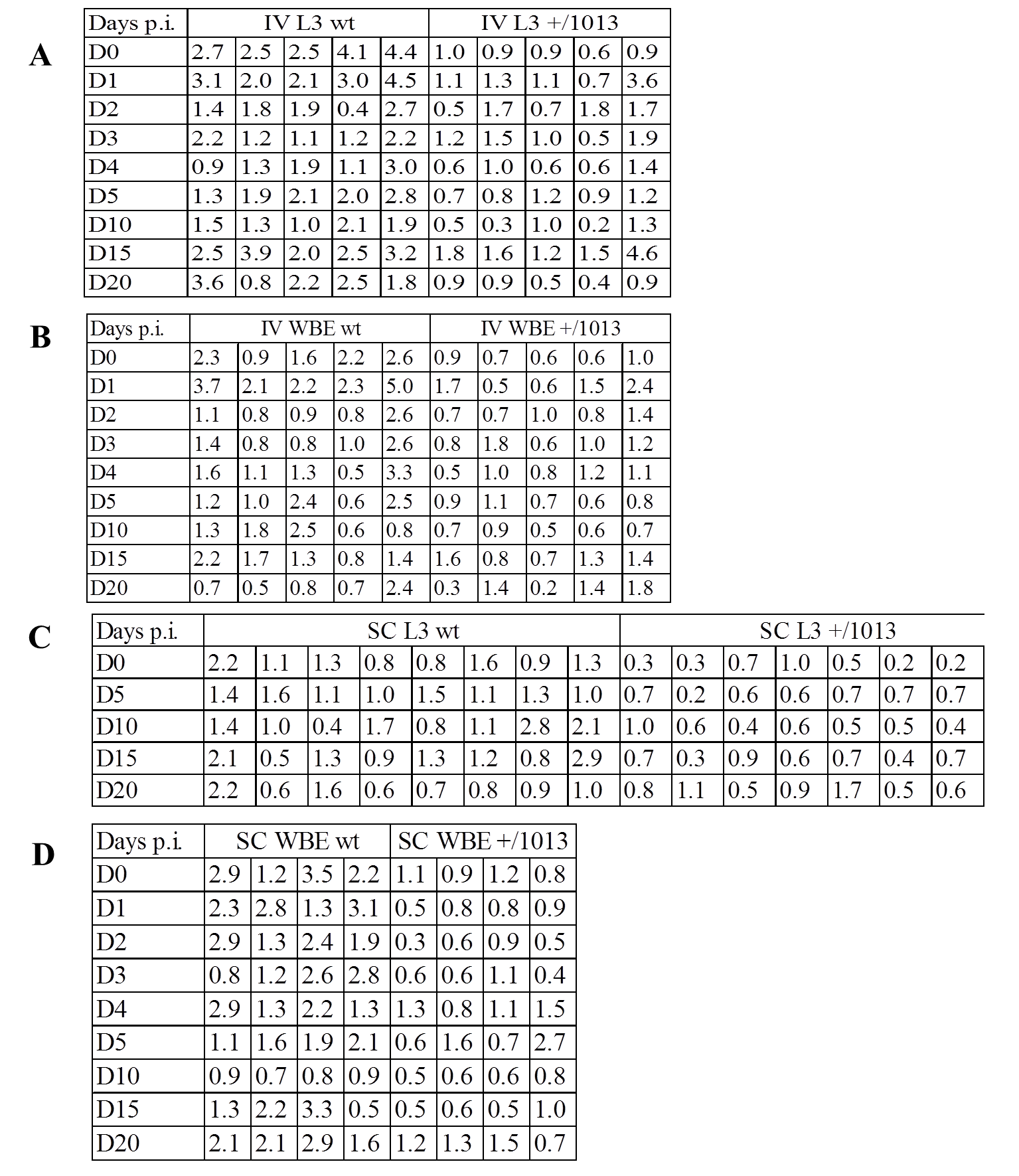

Supplement: S2 Table — Raw blood neutrophils counts from the experiments presented in Fig 2 panel C. Neutrophil counts (millions of cells per mL of blood) were obtained from mice either IV inoculated with 40 infective larvae (IV L3 wt and IV L3 +/1013, n = 5, panel A) or 10μg L3-derived whole body extracts (IV WBE wt and IV WBE +/1013, n = 5, panel B) or SC inoculated with 40 infective larvae (SC L3 wt and SC L3 +/1013, n = 8, panel C) or 10μg L3-derived whole body extracts (SC WBE wt and SC WBE +/1013, n = 4, panel D). (TIF) [file pntd.0004605.s010.tif]
